# Anthranilamide-based Short Peptides Self-Assembled Hydrogels as Antibacterial Agents

**DOI:** 10.1038/s41598-019-57342-6

**Published:** 2020-01-21

**Authors:** Vina R. Aldilla, Renxun Chen, Adam D. Martin, Christopher E. Marjo, Anne M. Rich, David StC. Black, Pall Thordarson, Naresh Kumar

**Affiliations:** 1 School of Chemistry, UNSW Sydney NSW, Sydney, 2052 Australia; 2 Dementia Research Centre, Faculty of Medicine and Health Sciences, Macquarie University, Sydney, NSW 2109 Australia; 3 Mark Wainwright Analytical Centre, UNSW Sydney, Sydney, NSW 2052 Australia

**Keywords:** Soft materials, Self-assembly

## Abstract

In this study, we describe the synthesis and molecular properties of anthranilamide-based short peptides which were synthesised *via* ring opening of isatoic anhydride in excellent yields. These short peptides were incorporated as low molecular weight gelators (LMWG), bola amphiphile, and *C*_3_-symmetric molecules to form hydrogels in low concentrations (0.07–0.30% (w/v)). The critical gel concentration (CGC), viscoelastic properties, secondary structure, and fibre morphology of these short peptides were influenced by the aromaticity of the capping group or by the presence of electronegative substituent (namely fluoro) and hydrophobic substituent (such as methyl) in the short peptides. In addition, the hydrogels showed antibacterial activity against *S*. *aureus* 38 and moderate toxicity against HEK cells *in vitro*.

## Introduction

Hydrogels, consisting of a large amount of water and an entangled fibrous network, are attractive materials which have been reported to have potential applications in many areas such as tissue engineering^1–4^, catalysis^5,6^, wound healing^7^, and cell culture^8^. Such hydrogels can be created through the self-assembly of small molecules, referred to as low molecular weight gelators (LMWG), forming a viscoelastic three-dimensional network that immobilizes water and results in hydrogel formation.

With their well-organized structure and diversity of amino acid functionality, peptides are one class of organic compounds that are generally suitable to be used as LMWG. Moreover, hydrogels made from peptides endow some fascinating advantages such as having lower toxicity, biodegradability, and non-immunogenic properties in most cases^9^. Recently, hydrogels made from short peptides are gaining attention due to their relative ease and low cost of synthesis, as compared to longer peptides. Diphenylalanine (FF), the core motif for the *ß*-amyloid self-assembling sequence^10^, is frequently incorporated as a backbone to generate short peptide based hydrogelators due to their ability to involve in extensive π-π stacking interactions^11^. Generally, an aromatic capping group is required to be appended on the *N-*terminus of the FF sequence, as this plays a pivotal role in enhancing intramolecular interactions which then can induce hydrogel formation. Fluorenylmethyloxycarbonyl (Fmoc), a protecting group widely used in solid phase synthesis (SPPS), is one of the most investigated capping group for generating short peptide-based hydrogelators^12–14^. Besides Fmoc, naphthalene^15,16^, indole^17^, benzimidazole^18^, or carbazole^19,20^ have also been reported to be employed as capping group to generate hydrogelators. In some cases, the selection of a capping group might have some influence on the physical and chemical properties of the resulting bulk hydrogels^18,21^.

Our previous work demonstrated that glyoxylamide-based short peptides, obtained *via* ring-opening reaction of *N*-acylisatin, can self-assemble to form organogels and hydrogels in low concentrations^22,23^. Even though hydrogels made from glyoxylamide-based short peptides exhibit high loading capacity of ciprofloxacin (an antibacterial agent) and sustainable release profile, they self-assemble over a relatively long time period^22^.

**Figure 1:**
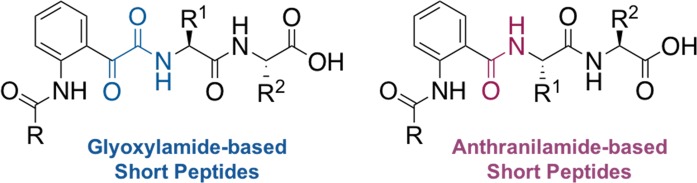
General structure of anthranilamide-based compared to glyoxylamide-based short peptides.

Anthranilamide, (2-AB), exhibits a similar structure to glyoxylamide (Fig. 1) and can be generated *via* ring-opening reaction of isatoic anhydride using a primary amine as a nucleophile^24^. Besides an aromatic group, which could enhance the π-π stacking interaction, anthranilamide also provides H-bonding sources that could participate in intermolecular interactions.

However, anthranilamide-appended short peptides have not been explored yet as an avenue to generate LMWG. In addition, peptide mimics bearing an anthranilamide moiety have been shown to exhibit antibacterial activity^25^. Therefore, in this work anthranilamide-based short peptides were synthesised and their ability to form antibacterial hydrogel through self-assembly, were investigated. These anthranilamide-based short peptides can also be generated in solution, which is beneficial in terms of scalability and access to chemical diversity. By changing the acyl group and introducing substituents (namely fluoro and methyl groups), the effect of hydrophobicity and electronegativity on the properties of the resulting hydrogel was investigated. In addition, anthranilamide-based short peptides were also incorporated in other self-assembled systems such as bola amphiphile (BA) and *C*_3_-symmetric molecules.

The characterization of these short peptides was conducted using various spectroscopy and microscopy techniques to investigate intermolecular interactions, secondary structure, and fibre morphology. In addition, rheology was conducted to examine the mechanical properties of the hydrogels formed. The antibacterial activity and cytotoxicity of the resulting hydrogel was measured *in vitro* against *S*. *aureus* 38 and HEK293T cells, respectively.

## Results and Discussions

### Synthesis of anthranilamide-based short peptides

**Figure 2:**
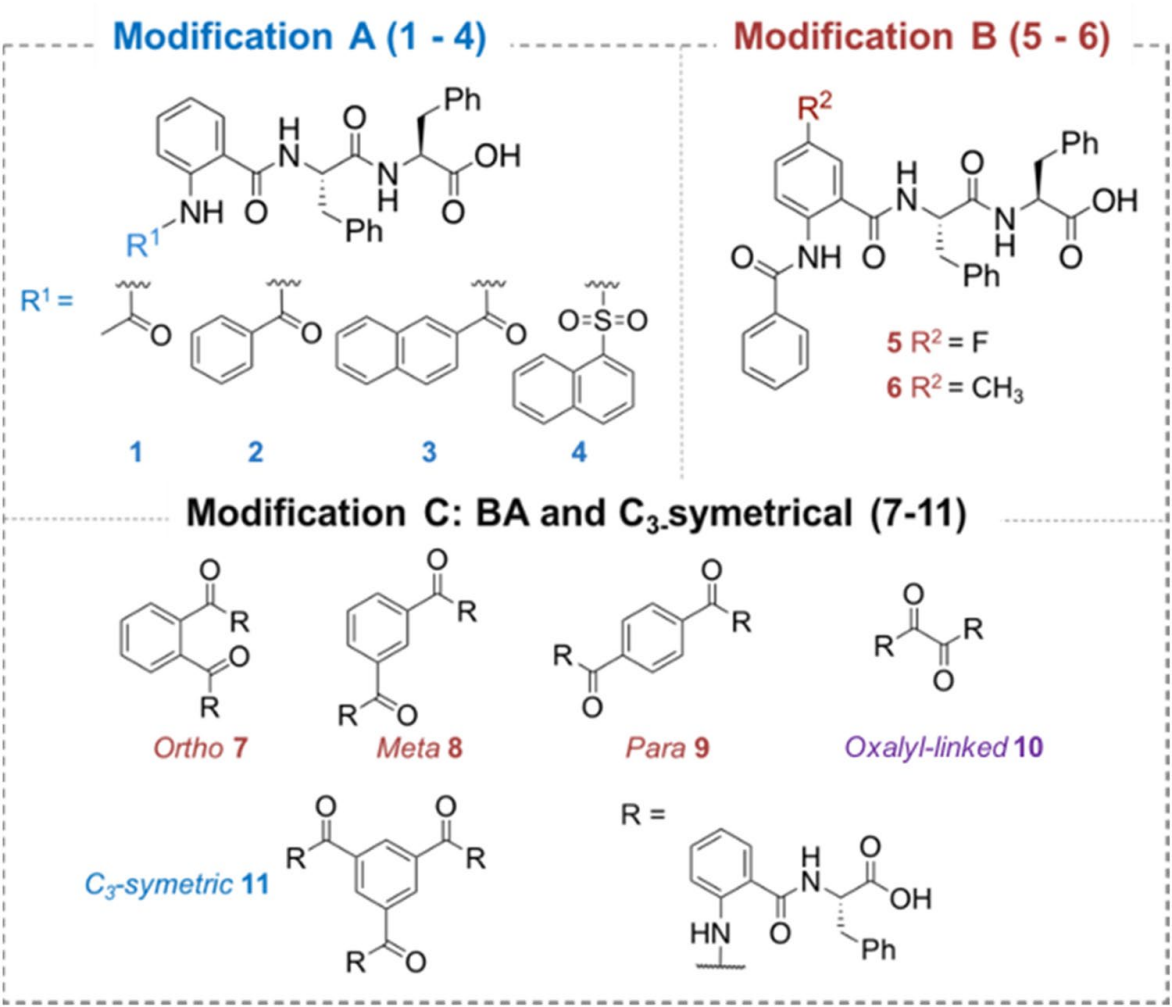
Structure of the modified anthranilamide-based hydrogelators 1–11.

The library of anthranilamide-based short peptides was designed based on three main modifications, which include modifying the acyl group R1 (modification A), introducing substituents at the 5 position of the capping group (modification B), and incorporation of anthranilamide-based short peptides into the bola amphiphile (BA) scaffold (modification C) Fig. 2.

A capping group of a short peptide-based hydrogelator could govern the stiffness and stability of the resulting hydrogels^18,26^. Therefore, hydrogels **1–4**, bearing different aromaticity on their capping groups, are envisaged to exhibit different properties. Fluorine, a small electronegative atom, and methyl were introduced (modification B) to investigate the effect of an electron-withdrawing or electron-donating group on the subsequent scaffolds, yielding compounds **5** and **6**, respectively.

Aside from conventional, linear low molecular weight gelators (LMWG), BA and *C*_3_-symmetric molecules comprise rather different molecular structures and are reported to have self-assembly properties^27–31^. Therefore, anthranilamide-based, short peptides were incorporated into BA and *C*_3_-symmetrical scaffolds. A Bola-amphiphile is an amphiphile which contains two hydrophilic ends that are connected *via* a hydrophobic spacer and which can cooperatively induce fibre formation^32–34^. The anthranilamide-based short peptides **7–9** were incorporated in different positions (*ortho-*, *meta-*, and *para-*) of benzene dicarboxamide to examine the effect of molecular structure on the properties of corresponding hydrogels. In addition, hydrogelators **10**, linked *via* an oxalyl spacer, were also synthesised to investigate the effect of using a shorter hydrophobic spacer.

Further building on the bola-amphiphile scaffolds **7**–**10** which contain two hydrophilic ends connected by a hydrophobic spacer, the anthranilamide-based peptides were incorporated into the *C*_3_ symmetric benzene-1,3,5-triscarboxamide (BTA)scaffold. BTA derivatives have been reported to form helical columnar stacks as the result of cooperative π-π stacking, which led to excellent hydrogelation^35^. Hence, the BTA-bridged anthranilamide-based short peptide **11** was expected to form a supramolecular hydrogel.

**Scheme 1:**
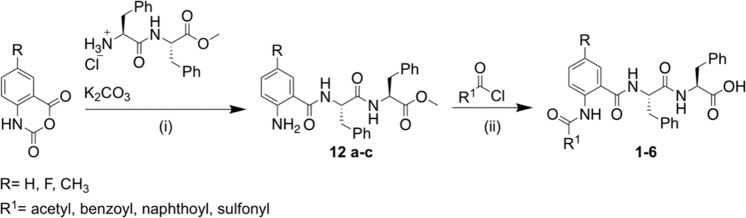
General scheme for ring-opening reaction of isatoic anhydride to obtain anthranilamide-based hydrogelators 1–6. (i) K_2_CO_3_ (2 equiv.), water, 60 °C, 18 h; (ii) base (1.5 equiv.), DCM, r.t, 4–18 h; (iii) LiOH (2.0 equiv.), THF: MeOH; H_2_O, r.t, 2–16 h.

Initially, to obtain anthranilamide-based short peptide, isatoic anhydride was ring-opened using a modified known procedure^36^. Isatoic anhydride was heated under reflux with methyl l-Phenylalanyl-l-phenylalaninate hydrochloride in the presence of an inorganic base to provide compounds **12a-c** as pure products after purification with column chromatography. Their structures were confirmed by ^1^H NMR as characteristic singlets around δ = 6.3 ppm and δ = 8.8 ppm corresponded to aniline and amide protons, respectively. Compounds **12a-c** were treated with the respective acyl or sulfonyl chloride followed by ester hydrolysis reaction to give the carboxylic acids **1–6** in excellent yield (70–83%) (Scheme 1).

**Scheme 2:**
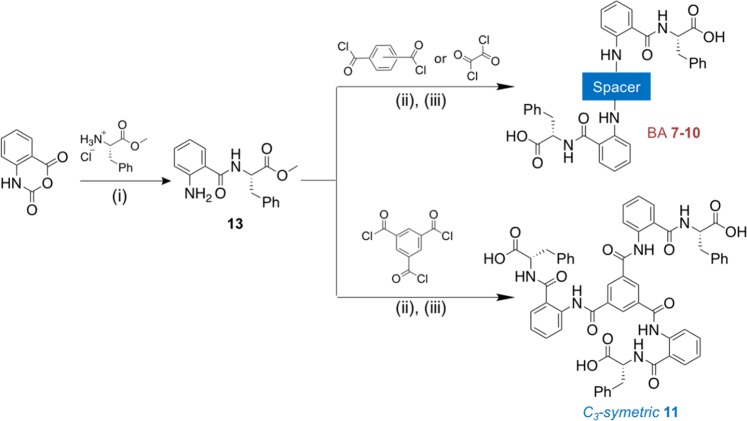
General synthetic scheme for BA 7–10 and C_3_ symmetric 11. (i) K_2_CO_3_ (2 equiv.), water, 60 °C, 18 h; (ii) Et_3_N (3.0 equiv.), DCM, r.t, 4–18 h; (iii) LiOH (2.0 equiv.), THF: MeOH; H_2_O, r.t, 2–16 h.

Similarly, the synthetic procedures to obtain BA **7–10** and *C*_3_ symmetric **11** derivatives are outlined in Scheme 2. Isatoic anhydride was ring-opened using one unit of l-phenylalanine to provide methyl (2-aminobenzoyl)- l-phenylalaninate **13**. After purification, **13** which was then reacted with the relevant acyl dichloride, oxalyl chloride, or trimesoyl chloride followed by hydrolysis to provide the final compounds **7–11** in good yield.

### Self-assembly of anthranilamide-based short peptides

Molecular self-assembly can be defined as a spontaneous process where disordered molecules or systems form more defined structures as result of intermolecular interactions^37^. In order to form a self-assembled gel, intermolecular interactions and balance between hydrophobicity and hydrophilicity of a short peptide-based gelators often play a significant role. An aromatic capping group of a short peptide-based hydrogel is usually designed in such a way that it could provide not only intermolecular interaction sources, but also a balance between hydrophobicity and hydrophilicity in the molecule. The partition coefficient, log P value, is a universal tool used to predict the hydrophobicity of a molecule. Having log P values between 2.53 and 5.25, which are considered to be ideal^38^, the anthranilamide-based hydrogelators **1–11** are expected to formed stable hydrogels. Additionally, the anthranilamide-based hydrogelator also provides a carbonyl group which might act as a hydrogen bond acceptor.

Several triggers such as physical stimuli, pH switch, solvent switch, or a combination of these, were employed to assess the self-assembly of compounds **1–11** in water. Initially, hydrogel formation of compounds **1–11**, at 1% (w/v), was investigated using a combination of pH switch and temperature switch. The compounds were heated in dilute sodium hydroxide (NaOH) in order to deprotonate the carboxylic acid group at the C-terminus of the short peptides. Upon cooling the solutions to room temperature, clear or opaque hydrogels with pH ranging from 9–12 were observed for *N*-acetyl **1**, *N-*benzoyl **2**, *N*-naphthoyl **3**, fluoro substituted **5** and methyl-substituted **6**, as shown in Fig. S1. The other compounds (**4**, **7–11**) remained as clear solutions even after 48 hours.

In an attempt to induce gelation in peptides which failed to form hydrogels using the previous method, glucono-δ-lactone (GdL) was added to basic solutions of **4** and **7**–**11**. Compared to a mineral acid, GdL promotes the formation of a homogenous hydrogel due to its slow hydrolysis and fast dissolution rate in water^39,40^. Addition of 3 equivalents of GdL, to protonate the C-terminus of the peptide, resulted in formation of translucent hydrogels for BA **8**–**10** (Table 1, Fig. S2).

**Table 1:** Critical Gel Concentration (CGC) and hydrogelation time of anthranilamide-based hydrogels formed using pH switch method.

Compounds		Trigger	Gelation time	Remarks^*a*^ (CGC^*b*^)	*Tg* (°C)	pH
*N*-acetyl **1**		NaOH + heat	<1 min	OG (0.3)	n.a.	9
*N-*benzoyl **2** ^*TR*^		NaOH + heat	30 min	CG (0.1)	76	11
*N*-naphthoyl **3** ^*TR*^		NaOH + heat	30 min	CG (0.1)	86	12
5-fluoro *N-*benzoyl **4** ^*TR*^		NaOH + heat	10 min	CG (0.07)	72	9
5-methyl *N-*benzoyl **6** ^*TR*^		NaOH + heat	15 min	CG (0.3)	56	9
Bola amphiphile	(*meta*-) **8**	pH switch and heat	24 h	CG (0.1)	n.a.	6.5
	(*para*-) **9**	pH switch and heat	24 h	CG (0.2)	n.a.	6.5
Oxalyl-linked **10**		pH switch and heat	24 h	CG (0.5)	n.a.	6.5

TR = thermoreversible. ^a^PG = partial gel; OG = opaque gel; CG: Clear gel.

^b^CGC = critical gel concentration (denoted as %w/v).

However, using this method, a white precipitate was observed from the *N*-naphthalene sulfonyl **4**, ortho **7**, and C_3_-symmetric **11**. In addition, the effect of salt which can induce gelation in basic peptide solutions^41^, due to high ionic was also investigated for peptides **1**–**11**. Addition of NaCl or CaCl_2_ to high pH solutions of compounds **1–11** resulted in precipitation of these short peptides, presumably due to a salting out process.

In a final attempt to induce gelation in peptides **4**, **7** and **11**, a solvent switch method using dimethyl sulfoxide (DMSO), methanol, or ethanol as co-solvent was employed^42–47^. The majority of the anthranilamide-based short peptides formed a turbid solution which then clarified to various degrees, over the course of seconds to minutes, and subsequently formed clear or opaque hydrogels (Table S1, Fig. S2). However, gels were observed only for the first 5 minutes for *N*-naphthalene sulfonyl **4** and BA **7** (*ortho-*), as water and a white precipitate was observed to evolve over time when using DMSO: water at 1% w/v (Fig. S4). Peptide **11** formed a precipitate for all solvent switch conditions tested. Given that these hydrogels were designed with antibacterial applications in mind, due to the potential toxicity concerns arising from the use of organic co-solvents, further characterisation of peptides **4**, **7** and **11** was not carried out.

The critical gel concentration (CGC) represents the minimum amount of the anthranilamide-based short peptide required to form a hydrogel. The CGC of anthranilamide-based short peptides were qualitatively assessed by varying the short peptide concentrations and conducting vial inversion test^48–50^. Hydrogels composed of anthranilamide-based short peptides exhibit relatively low CGCs ranging from 0.07–0.30% (w/v) with gelation achieved either through combinations of pH and temperature switch (Table 1). Here, gelation time represents the time required for these short peptides, at 1% (w/v), to form self-supporting hydrogels as assessed through the vial inversion test.

### Characterisation of anthranilamide-based hydrogels

It has been reported that interaction between aromatic units, in short peptide hydrogelators, plays a prominent role for self-assembly to occur^51,52^. Therefore, the π-π stacking interaction between aromatic groups of anthranilamide-based short peptides was investigated using ^1^H NMR, UV-Vis, and Circular Dichroism (CD) spectroscopy.

**Figure 3:**
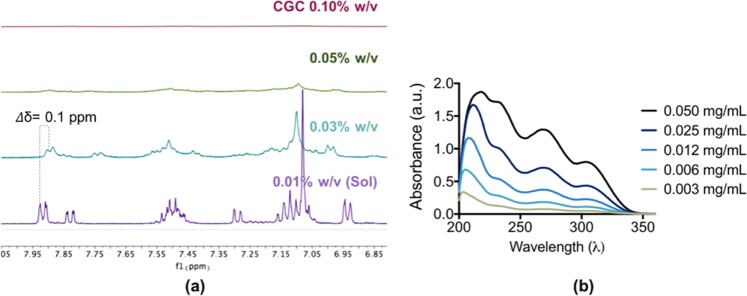
(**a**) 1H NMR and (**b**) UV-Vis spectra of *N*-benzoyl **2** at different concentrations.

^***1***^***H NMR and UV-Vis*** To investigate the role of the π-π stacking interactions, concentration dependent ^1^H NMR studies were performed on *N-*acetyl **1**, *N-*benzoyl **2**, and *N-*naphthoyl **3**, bearing different aromatic cap. Upon increasing the concentration of these hydrogelators, notable up-field chemical shifts (𝛥δ = 0.1 ppm) were observed for the aryl proton from the aromatic cap which indicated their involvement in π- π stacking that initiate self-assembly process (Figs. 3a and S5)^53,54^. The peak broadening (Fig. 3a, green) indicated that the self-assembly of short peptides **1–3** started to occur at very low concentrations. However, complete transformation from solution phase into gel phase was observed at their CGC, as indicated by disappearance of the ^1^H NMR features (Figs. 3a and S5, red)^55^.

To support the ^1^H NMR analysis, concentration dependent UV-Vis was carried out. UV-Vis absorption of *N-*benzoyl **2** showed a bathochromic shift (from 203 nm − 217 nm) and enhancement of a shoulder peak ranging from 240 nm – 350 nm as the concentration was increased from 0.003 mg mL^−1^ to 0.050 mg mL^−1^ (Fig. 3b). Similarly, *N-*acetyl **1** (bearing less aromatic cap) and *N-*naphthoyl **3** (bearing a more aromatic cap) also exhibited bathochromic shifts from 202 nm − 213 nm and enhancement of shoulder peaks ranging from 240–350 nm (Fig. S6). These results further support the observation that aromatic groups promote the self-assembly of anthranilamide-based short peptides to form well defined nanofibres leading to hydrogel formation.

**Figure 4:**
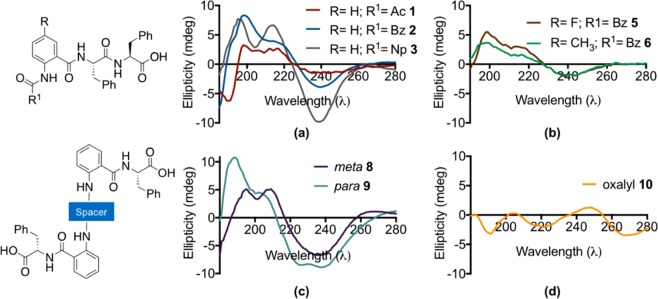
CD spectra of (**a**) compounds 1–3; (**b**) compounds 4 and 5; (**c**) BA 8–9; and (**d**) BA linked via an oxalyl linker 10.

***Circular Dichroism (CD) spectroscopy and ATR-FTIR*** Peptides often exhibit conformational motifs such as *a*-helix, *ß*-sheets, or disordered coils which can be determined using CD spectra and FTIR spectroscopy. Initially, the far UV region (240 nm – 190 nm) where the main absorbing group is a peptide bond, was investigated using a CD spectrophotometer. The negative band at ~195 nm and relatively low ellipticity above 210 nm observed for *N-*acetyl **1** indicated formation of a random coil secondary structure^56^. On the other hand, the *N-*benzoyl **2** and *N-*naphthoyl **3** possessed *ß*-sheet secondary structures as indicated by the presence of positive maxima at around 190–200 nm (π → π*) and negative minima around 230 nm (n → π*) (Fig. 4a)^57^. The presence of two positive maxima, at 197 nm and 220 nm, for **2** and **3** suggested formation of dipeptide nanotubes which are rich in *ß*-sheet secondary structure facilitated through π-π stacking interactions from the aromatic capping group^58,59^.

Anthranilamide-based short peptides **5** and **6**, bearing fluoro and methyl as substituents, exhibit similar CD patterns to those of *N-*benzoyl **2** (Fig. 4b), which suggest that introduction of a substituent at the 5-position of anthranilamide-based short peptides does not affect their secondary structure (Fig. 4c,d).

*ß*-sheet secondary structure was also observed from dilute solutions of both BA **8** (*meta-*) and **9** (*para*-). On the other hand, BA **10** (linked *via* an oxalyl linker) showed a characteristic disordered coil by appearance of a strong negative band below 200 nm and a weak positive band at ~218 nm^60^.

**Figure 5:**
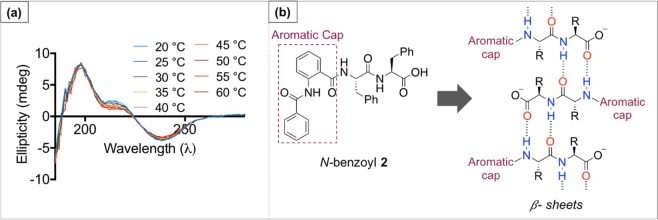
(**a**) Temperature dependent CD spectra of *N*-benzoyl 2. (**b**) Proposed *ß*-sheet arrangements observed from *N*-benzoyl 2 using CD spectroscopy and FTIR.

In addition, CD spectroscopy was also used as a tool to investigate gradual thermal denaturation of anthranilamide-based short peptides. Despite the CD signal of *N-*benzoyl **2** (as model compound) being gradually decreased, the overall *ß*-sheet feature was preserved upon increasing temperature from 25 °C to 60 °C. This result indicates that there are no significant conformational changes and demonstrates thermal stability of *N-*benzoyl **2** under physiological conditions (Fig. 5), which is important to note for future antimicrobial and cytotoxicity studies.

FTIR was used to further confirm the formation of either *ß*-sheet structure or disordered coil in anthranilamide-based hydrogel **1–10** (Figs. S7–14). The amide I region of D_2_O gels made from *N-*benzoyl **2**, *N-*naphthoyl **3**, and BA **8–9** exhibited peaks that correspond to *ß*-sheet structure (1625 cm^−1^ −1640 cm^−1^)^57^. Meanwhile, hydrogels made from *N-*acetyl **1** and BA **10** showed peaks at 1647 cm^−1^ and 1640 cm^−1^, respectively, which support their disordered coil structure observed by CD spectroscopy^61,62^. In addition, peaks correspond to their respective secondary structure were also observed in xerogels (air-dried H_2_O gel) of these anthranilamide-based short peptides **1–10** (Figs. S7b–S14b). This suggested that secondary structures of these hydrogels were retained, regardless of whether the peptide was in a lyophilized or hydrated environment^63^.

### Mechanical properties

Mechanical properties of hydrogelators prepared from anthranilamide-based short peptides were investigated using a rheometer. All hydrogels, except those composed of **6** displayed frequency-independent behavior during frequency sweep tests (FST) (Fig. S15). Hydrogels composed of **6** appeared to undergo irreversible deformation at frequencies >2 Hz, indicating that the gel network is metastable^58,59^.

The stiffness of a hydrogel can be approximated by its Gʹ value, where a higher value corresponds to a stiffer hydrogel^60^. It can be seen that changing the capping group from *N-*acetyl to *N-*benzoyl or *N-*naphthoyl significantly increases the stiffness of the resulting hydrogel from 3.4 kPa to 16.9 and 5.7 kPa, respectively (Table 2). This result might be ascribed to enhanced aromatic or hydrophobic interactions due to *N-*benzoyl **2** and *N*-naphthoyl **3** capping groups.

**Table 2:** Modulus storage (Gʹ), modulus loss (Gʹʹ), and linear viscoelastic region (LVER) of hydrogels 1–10 obtained from frequency sweep and strain sweep test.

	Gʹ (kPa)	Gʹʹ (kPa)	LVER (%)		Gʹ (kPa)	Gʹʹ (kPa)	LVER (%)
*N*-acetyl **1**	3.40	0.97	0.2 ± 0.01	BA **8** (*meta*)	3.13	0.16	5.2 ± 0.04
*N*-benzoyl **2**	16.93	1.16	5.6 ± 0.03	BA **9** (*para*)	22.70	1.12	4.7 ± 0.05
*N-*naphthoyl **3**	5.74	0.53	1.9 ± 0.02	BA **10**	10.39	0.84	1.5 ± 0.02
Fluoro **5**	11.39	0.75	8.5 ± 0.02				
Methyl 6^*^	0.12	0.012	0.6 ± 0.01				

^*^Gʹʹ ≥ Gʹ at f ≥ 4.5 Hz.

Hydrogel **5** (with a fluoro substituent), also showed characteristics of a stable hydrogel with similar stiffness (11.4 kPa) to *N-*benzoyl **2**, indicating that the installation of an electron-withdrawing fluoro group at this position does not affect the overall mechanical properties of the hydrogel. In contrast, hydrogel **6** bearing an electron-donating methyl substituent showed a notable decrease in strength with Gʹ = 120 Pa (Table 2). Hydrogel **6** showed frequency-dependent behaviour at frequencies above 2 Hz, where hydrogel rupture ultimately occurred. At frequencies below 1 Hz, however, frequency independent behaviour was observed.

For the bola amphiphile-type peptides, depending on the spacer, diverse mechanical properties were observed for hydrogel **8–10** owing to their different self-assembled structures. BA **8**, which is linked at the *meta-* position, gives a moderately stiff hydrogel with Gʹ = 1.6 kPa. Interestingly, the structural isomer (*para-* **9**) yields a stiffer hydrogel (Gʹ = 12.9 kPa), possibly due to a more linear packing motif which could stabilize the multilayer nano-structure. In addition, BA connected through an oxalyl spacer **10** also showed characteristics of stable hydrogels with Gʹ = 10.3 kPa.

The strain sweep test (SST) was conducted to determine the linear viscoelastic region (LVER) of a hydrogel. A larger LVER suggests that the hydrogel is more resistant to an applied oscillatory strain, such as that which can be applied by cells^64^. Hydrogels composed of *N-*acetyl capped peptide **1** show deformation upon the application of a relatively small strain (0.2 ± 0.01%), indicating its unstable nature, potentially due to a lack of aromatic/hydrophobic interactions^65^. Aromatic – aromatic interactions are known to form more stable supramolecular hydrogels^66^. In agreement, the *N-*benzoyl **2** and *N-*naphthoyl **3** were more amenable to applied strains, with LVERs up to 5.6 ± 0.03% and 1.9 ± 0.02%, respectively (Fig. S16).

The presence of fluoro, as an electron-withdrawing substituent, in hydrogel **5** increased the LVER from 5.6% to 8.5%. In contrast, hydrogel **6**, bearing a methyl substituent, exhibited a significantly shorter LVER (0.6%). This result might be accounted for by the electronic contributions from the substituent on the anthranilamide-core, which affected the overall mechanical properties of the resulting hydrogels.

Compared to BA **8** (*meta-*), the isomeric structure BA **9** (*para-*) exhibited a shorter LVER, presumably due to its more linear packing which reduces the flexibility of the resulting hydrogels. In addition, BA **10** (*oxalyl-*) displayed a significantly shorter LVER, potentially due to the less aromatic and shorter spacer compared to BA **8** and **9**.

### Network morphology of the self-assembled gels

**Figure 6:**
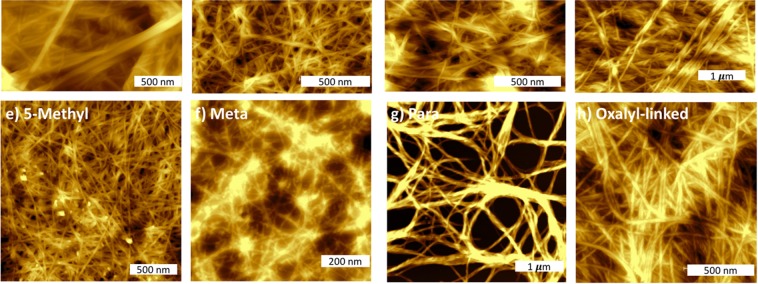
AFM image of fibre networks obtained from xerogel of anthranilamide-based hydrogelators 1–11 at four time below their CGC. Xerogel made from (**a**) N-acetyl 1 at 0.08%w/v, (**b**) N-benzoyl 2 at 0.03%w/v, (**c**) N-naphthoyl 3 at 0.03%w/v, (**d**) fluoro 5 at 0.02% w/v, (**e**) methyl 6 at 0.08%w/v, (**f**) BA 8 (meta-) at 0.03%, (**g**) BA 9 (para-) at 0.03%, and (**h**) BA 10 (linked via oxalyl) at 0.13%w/v.

To gain some insight into the morphology of the hydrogels, xerogels of **1–3**, **5–6**, and **8–10** were imaged using atomic force microscopy (AFM). In general, these hydrogels possessed fibre-like structures with different diameters as shown in Fig. 6. *N-*acetyl bearing hydrogel **1** consisted of two fibre populations, small fibres with diameter of 60 ± 13 nm and larger fibres with diameter of 180 ± 20 nm (Fig. 6a). Straight fibres with no junction zones were observed and might explain the brittle characteristics of these hydrogels as measured by rheology. It has been reported that less aromatic hydrogelators tend to form thicker fibres than more aromatic hydrogelators^17,18^. Consistently, *N-*benzoyl **2** and *N-*naphthoyl **3** (bearing more aromatic groups) exhibit fibres with smaller diameters of 18 ± 5 nm and 12 ± 5 nm, respectively.

Introducing substituents, such as fluoro or methyl, did not significantly change the overall morphology of the fibres (Fig. 6d–e). The fluoro substituted hydrogel **5** possessed fibres with similar diameter (16 ± 0.1 nm) to those of hydrogel **2**. Meanwhile, hydrogel **6** (having a methyl substituent) showed a notable decrease in diameter (9 ± 0.1 nm). This is somewhat surprising, given the significant differences observed in the mechanical properties (storage modulus and LVER) for hydrogels of **5** and **6**.

In addition to exhibiting distinct mechanical properties, the isomeric peptides BA **8** (*meta-*) and BA **9** (*para-*) also showed divergent fibre morphology (Fig. 6f–g). Hydrogel **8** (*meta-*), showed formation of small straight fibres (15 ± 2 nm), while the hydrogel made from *para-* **9** displayed twisted fibres with diameter of 112 ± 10 nm. The formation of thicker fibres along with extensive bundling, observed from BA **9** (*para-*), clarify their much higher Gʹ value compared to BA **8** (*meta-*) shown in rheology^67^. In addition, BA **10** (linked *via* an oxalyl spacer) showed formation of slightly curved fibres with diameter of 29 ± 7 nm.

### Antibacterial activity of the anthranilamide-based hydrogels

**Figure 7:**
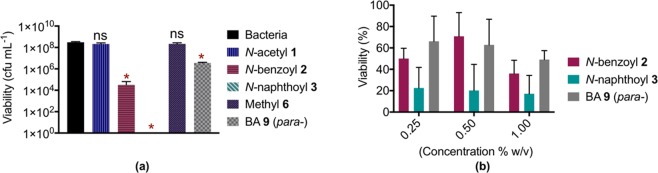
(**a**) Antibacterial activity against S. aureus 38 using viable count method (n = 2, *p < 0.001, ns = no-significant difference between hydrogels and negative control) of hydrogels made from N-acetyl 1, N-benzoyl 2, N-naphthoyl 3, methyl 6, and BA 9 (para-) at 1% w/v. (**b**) Cytotoxicity of hydrogels made from N-benzoyl 2, N-naphthoyl 3, and BA 9 (para-) against HEK293T cells.

With a complete understanding of the hydrogel ultrastructure now in-hand, the antibacterial activity of *N*-acetyl **1**, *N*-benzoyl **2** and *N*-naphthoyl **3** was assessed, in order to evaluate the effect of increased hydrophobicity on antibacterial activity. Further, the stiffest (BA **9**) and softest (methyl **6**) of the remaining gelators were tested to discern the relationship of hydrogel stiffness to antimicrobial activity. *Staphylococcus aureus*, the most common causative organism for skin and soft tissue infections, was chosen as the bacterial strain to assess the hydrogels antibacterial activity^68^. These hydrogel surfaces was challenged with bacterial inoculum of 3 × 10^4^ cfu mL^−1^ and viability was measured after 18 hour of incubation. Interestingly, *N*-benzoyl **2**, *N-*naphthoyl **3**, and BA **9** (*para-*) exhibited significant bacteria activity with bacteria reduction of 4.4 Log_10_, 9.0 Log_10_, and 1.9 Log_10_, respectively (Fig. 7a). In contrast, the less stiff hydrogels (*N*-acetyl **1** and methyl **6)** did not show significant bacterial reduction. This result was anticipated, as rheological properties (in particular higher Gʹ value) were reported to give rise to antibacterial function of a hydrogel by providing mechanical support to individual fibres and fibrous networks^69^. Furthermore, the observed antibacterial activity was not due to the presence of NaOH as the concentration of NaOH in all of the hydrogel tested were found to be below their MIC against *S*. *aureus* (7.5 mg/mL)^70^. This results is consistent with previous studies, where short peptide-based hydrogels without cationiccharge, were shown to exhibit antibacterial activity against Gram positive and Gram negative bacteria^71–74^.

### Cytotoxicity of hydrogels 2, 3, and 9

In order to successfully treat infection, antimicrobial compounds need to exhibit high toxicity towards bacteria but low toxicity towards mammalian cells. Although the diphenylalanine moiety is known to be non-toxic, the cytotoxicity of short peptide based hydrogelators are often defined by their capping group^19,75–77^.

With this in mind, the cytotoxicity of lead candidates *N-*benzoyl **2**, *N-*naphthoyl **3**, and BA **9** (para) from the antibacterial studies were examined against HEK293T cells, as a robust mammalian cell model. As the antibacterial activity of the **2**, **3** and **9** was evaluated in the gel phase, contact cytotoxicity of hydrogels at concentrations of 0.25, 0.5, and 1% (w/v) was examined. At these concentrations, which are far above their CGC, both **2** and **9** exhibited moderate cytotoxicity towards HEK cells (Fig. 7b) with no significant variation upon concentrations. Unfortunately, the *N*-naphthoyl hydrogel **3** showed poor cell viability against HEK cells (Fig. 7b, green), potentially owing to its increased hydrophobicity, which has previously been shown to correlate with cytotoxicity for short peptide gelators^78^.

## Conclusions

Anthranilamide-based short peptides have been successfully incorporated as LMWG, bola amphiphile (BA), and *C*_3_ symmetric molecule *via* ring-opening reactions of isatoic anhydride in solution phase with excellent yield. The short peptides reported herein formed hydrogels using combinations of pH switch and heat or solvent switch as a trigger in relatively low concentrations (0.07–0.30%). The hydrogel properties (such as mechanical strength, secondary structure, and fibre morphology) can be modulated by varying hydrophobicity or introducing substituents on the capping group. Hydrogels made from *N-*benzoyl **2** and BA **9** showed most favorable viscoelastic properties. In addition, these hydrogels exhibit antibacterial activity against *S*. *aureus* and moderate toxicity against HEK cells. Further modification to the hydrogel scaffold is required to improve the cytotoxicity of the hydrogels for biomedical applications, such as topical antibacterial gels.

## Materials and Methods

### Synthesis

All chemicals and solvents used were purchased from Chemimpex, Combi Blocks, or Sigma Aldrich and were used without any further purification.

The anthranilamide short peptides were synthesised *via* ring opening reactions of isatoic anhydride. The detailed synthetic procedure for hydrogelators **1–11** along with their characterization data (IR, ^1^H NMR, ^13^C NMR, and HRMS) are given in Supplementary materials.

### Preparation of hydrogels

To a pre-weighed compound in a glass vial, 1 M NaOH (2–8 molar equiv.) was added followed by addition of Mili-Q water. The suspension was then heated and vortexed vigorously for 15 minutes to completely dissolve the compound. In the case of *N-*acetyl **1**, *N-*benzoyl **2**, N-naphthoyl **3**, fluoro **5** and methyl **6**, these clear solutions were left to cool to room temperature for 1–30 minutes. GdL (2–3 equiv.) was added to a solution of compounds **7–11** in NaOH. The turbid solution was left at room temperature for 24 hours to form a clear hydrogel. The vials were then inverted and allowed to stand overnight to confirm their hydrogel formation. Critical gel concentration, shown as a percentage, indicates the lowest mass of hydrogelator where the self-supporting characteristic is still observed divided by the total volume of the hydrogel^22^.

Hydrogel formation *via* solvent switch method has been previously described^47^. Similarly, compounds **1–9** was dissolved in 20% and 50% of either DMSO, ethanol, or methanol. Subsequently, respective amount of Mili-Q water was added to the solutions of **1–9** followed by gentle mixing.

### ^1^H NMR

The NMR samples was prepared by dissolving *N-*acetyl **1** in an equivalent of NaOD, which followed by addition of respective amount of D_2_O to make up final concentration of 0.04%, 0.08%, 0.15%, and 0.3% (w/v). Meanwhile, *N-*benzoyl **2** and *N-*naphthoyl **3** were dissolved in 5 and 8 equivalents of NaOD, respectively, followed by addition of respective amount of D_2_O to make up final solutions of 0.01%, 0.03%, 0.05%, and 0.10% (w/v). ^1^H NMR spectra were recorded using a Bruker Avance III 400 MHz NMR spectrometer. The NMR spectra were processed using the Bruker TOPSPIN 3.0 software.

### CD Spectroscopy

The anthranilamide-based hydrogels were prepared at 0.8% (w/v) and were diluted 10 times before being transferred into a 0.5 mm path length cuvette. Meanwhile, for the concentration dependence, the hydrogel made from *N-*benzoyl **2** at 0.8% (w/v) was diluted to make up concentrations ranging from 0.058 mM – 0.470 mM before being transferred into a 0.5 mm path length cuvette.

CD spectrum were obtained using a ChirascanPlus CD spectrometer (Applied Photophysics, UK) scanning wavelengths of 180–500 nm with a bandwidth of 1 nm, 0.6 s per point, and step of 1 nm^22^. The outcome of three experiments were then averaged and plotted into a single plot value. The high tension (HT) value of each experiment was maintained to be below 600 mV (Fig. S17).

### UV-Vis Spectroscopy

The UV-Vis spectra of *N-*acetyl **1**, *N-*benzoyl **2**, *N-*naphthoyl **3** were measured using Agilent Cary 60 UV-Vis with concentrations ranging from 0.003 mg mL^−1^ to 0.050 mg mL^−1^.

### Attenuated total reflectance fourier-transform infrared spectroscopy (ATR-FTIR)

D_2_O gels of anthranilamide-based short peptides **1–10** were pre-formed in a glass vial at 3%w/v. Heat was applied to these D_2_O gels to trigger the transformation to their solution-phase. Subsequently, two drops of each D_2_O gels was placed on the ATR crystal and allowed to stand for 5 minutes. On the other hand, xerogels were formed *in-situ* by applying nitrogen to two droplets of hydrogel made from anthranilamide-based short peptides **1–10** at 3% (w/v). The spectrum was recorded using a Spectrum 100 FTIR spectrometer (PerkinElmer, USA) fitted with a 1 mm diamond-ZnSe crystal from 4000–650 cm^−1^ with 4 cm^−1^ resolutions and 32 scans.

### Rheology measurements

The viscoelastic properties of hydrogels made from anthranilamide-based short peptides were determined using Anton Paar MCR 302 rheometer with a 25 mm stainless steel parallel plate configuration, as previously described^22,23^. Pre-formed hydrogel was warmed, using a heat gun, and the resulting solution (560 µL of 1% (w/v)) was transferred onto the rheometer plate. The other plate was lowered to its measuring position, 1 mm gap, and the solution was allowed to stand for 2–24 h for the gel to completely form. To prevent solvent evaporation, a solvent trap using Mili-Q water and a Peltier temperature controller hood was employed. The frequency sweep test (FST) was performed at constant strain of 0.1% using frequency of 10 Hz to 0.01 Hz. Meanwhile, strain sweep test (SST) was conducted at constant frequency of 1 Hz using 0.1% to 100% strain. In addition, a temperature sweep test was examined to determine *Tg* values using constant frequency of 1 Hz and constant strain of 0.1% with temperature ramping from 25 °C to 90 °C. The rheology data presented are an average of three repeats.

### Atomic force microscopy

A drop of these pre-gel solution was casted onto a mica substrate and the droplet was gently spread using a glass slide. Pre-gel solution of anthranilamide-based short peptides were obtained by either heating the thermo-reversible hydrogels **1–3** and **5–6**, or quickly transferring solutions of **8**–**10** after GdL addition but prior to gelation. The samples were left to dry overnight before being imaged. Imaging was undertaken on a Bruker Multimode 8 Atomic Force Microscope in Scanasyst Air (PeakForce Tappings) mode, which is based on tapping mode AFM, but whereby the imaging parameters are constantly optimized through the force curves that are collected, preventing damage of soft samples^22^. Bruker Scanasyst-Air probes with a spring constant of 0.4–0.8 N m^−1^ and a tip radius of 2 nm were used in this experiment.

### Antibacterial activity

Antibacterial activity of the anthranilamide-based hydrogels were performed using modification of a known method^79^. Initially, a single colony of *S*. *aureus* 38 was grown overnight in Luria-Bertani (LB) broth medium (Sigma-Aldrich) at 37 °C. The resulting bacteria culture was centrifuged and harvested. The bacteria pellet was re-suspended in the same volume of LB, twice. Optical density (OD) of the resulting culture was adjusted to 0.1 at 600 nm in LB (10^8^ cfu mL^−1^) and the bacterial solution was further adjusted to 3 × 10^4^ cfu mL^−1^. The bacteria solution (1 mL) was carefully casted on top of the pre-formed hydrogels (1% w/v; 1 mL) in glass vials. As a negative control, bacteria solution without a hydrogel was used in the experiment. These vials were incubated at 37 °C for 18 h. Serial dilution was performed to the 100 µL of the bacteria solution (on top of the hydrogels) using phosphate-buffered saline (PBS). 20 µL of each dilution, were carefully transferred into nutrient agar plates and incubated at 37 °C for another 18 h. The following day, bacterial growth inhibitions were recorded using viable count methods. This experiment was performed twice in triplicate. Multiple sample comparison was performed using one-way ANOVA at *p* < *0*.*05*.

### Cytotoxicity assays

Similar to previously reported method^22^, cytotoxicity measurement was performed using an Alamar Blue colorimetric assay on HEK293T cells. HEK293T cells were passaged using standard cell culture procedures. Cells were detached with trypsin and centrifuged (1000 rpm for 3 min). After supernatant was removed, the cells were re-suspended in Dulbecco’s Modified Eagle Medium (DMEM) at a concentration of 60,000 cells/mL. Cells were seeded onto hydrogels at a concentration of 6,000 cells/well. Hydrogels were prepared as described above and 100 µL cast into the wells of a 96 well plate in triplicate. After incubating overnight, 100 µL media was added to the set hydrogels and incubated overnight. The following day, excess media was aspirated and cells seeded atop the hydrogels as above. After incubation for 24 hours, 10 µL Alamar Blue was added to each well, followed by further incubation for another 4 hours. Wells containing cell-free hydrogels, no hydrogel substrate, and a negative control of 20% (v/v) DMSO were prepared as controls. BioRad Benchmark plate reader was used to measure the absorbance at 570 nm and 596 nm. Each experiment was repeated at least three times.

## Supplementary information

Supporting Information.
