# Supplementary material for: Anthranilamide-based Short Peptides Self-Assembled Hydrogels as Antibacterial Agents
Source: Sci Rep. 2020 Jan 21;10:770. doi: 10.1038/s41598-019-57342-6 (PMC6972728; doi:10.1038/s41598-019-57342-6)
Supplement: Supplementary file 1 — Supporting Information. [file 41598_2019_57342_MOESM1_ESM.pdf]

Anthranilamide-based Short Peptides Self-Assembled Hydrogels as Antibacterial Agents

Vina R. Aldilla[1], Renxun Chen[1], Adam D. Martin[2,*], Christopher E. Marjo[3], Anne M. Rich[3], David StC. Black[1], Pall Thordarson[1], and Naresh Kumar[1,*]

[1]School of Chemistry, UNSW Sydney, NSW 2052, Australia
[2]Dementia Research Centre, Faculty of Medicine and Health Sciences, Macquarie University, Sydney, NSW 2109, Australia
[3]Mark Wainwright Analytical Centre, UNSW Sydney, NSW 2052, Australia
[*]n.kumar@unsw.edu.au, adam.martin@mq.edu.au

Table S 1 Hydrogelation of anthranilamide-based peptide mimics **1-11** using solvent switch method.

Compounds	Observation[a] (gelation time)[b]		
	DMSO : H_2O	EtOH : H_2O	MeOH : H_2O
N-acetyl **1**	PG (immed)	OG (immed)	OG (immed)
N-benzoyl **2**	CG (10 min)	CG (10 min)	CG (10 min)
N-naphthoyl **3**	CG (immed)	OG (immed)	CG (immed)
N-naphthsulfo **4**	OG* → P	P	P
5-fluoro *N*-benzoyl **5**	CG (10 min)	OG (immed)	OG (immed)
5-methyl *N*-benzoyl **6**	CG (immed)	CG (immed)	CG (immed)
Bola amphiphile *Ortho* **7**	OG* → P	P	P
Bola amphiphile *meta* **8**	CG (5 min)	CG (immed)	CG (immed)
Bola amphiphile *para* **9**	OG (immed)	PG	PG
Oxalyl-linked **10**	OG (immed)	P	P
C_3 symetric **11**	CG (3hours)	P	P

[a] PG= partial gel; OG= opaque gel; CG: Clear gel; P= precipitate
[b] immed= immediately; *in the first 5 minutes

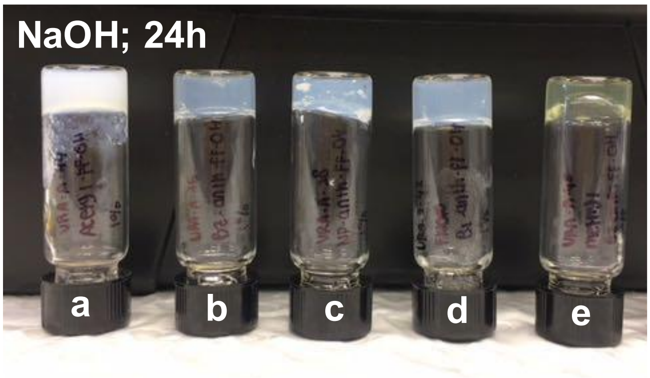

Figure S 1 (a) Opaque hydrogel observed from *N*-acetyl **1** and clear hydrogels observed from (b) *N*-benzoyl **2**, (c) *N*-naphthoyl **3**, (d) fluoro **5**, and (e) methyl **6**. Image was taken after vial inversion test was conducted for 24h

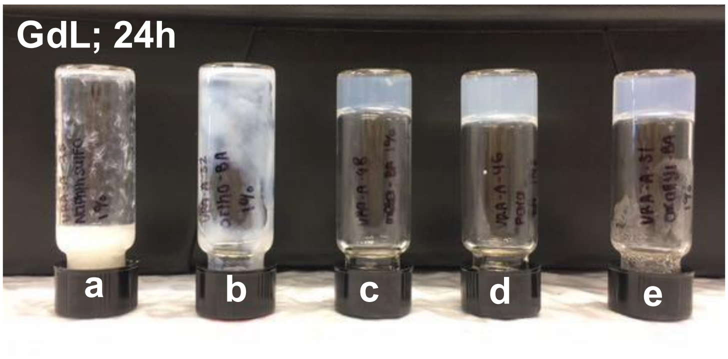

Figure S 2 Precipitate observed from (a) *N*-naphthalene sulfonyl **4** and (b) BA **7** *(ortho-)* at 1 %w/v after addition of GdL. Meanwhile, clear hydrogels observed from (c) BA **8** (*meta*-), (d) BA **9** (*para*-), and (e) oxallyl **10** at 1 %w/v using similar treatment. Image was taken after vial inversion test was conducted for 24h.

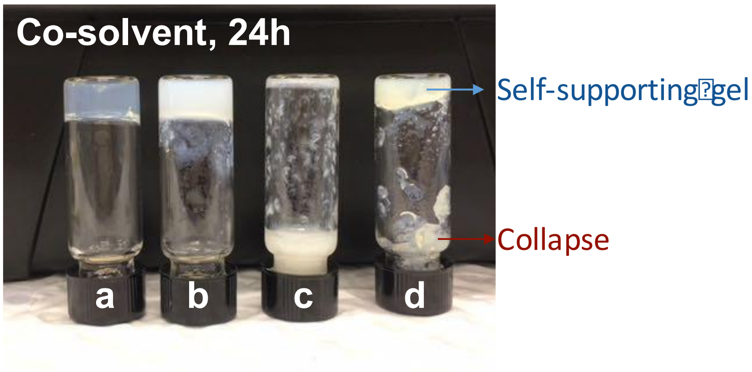

Figure S 3 Representative example showing the appearance of (a) clear gel obtained from *N*-benzoyl **2**; (b) opaque gel obtained from *N*-naphthoyl **3**; (c) precipitate observed from *N*-naphthalene sulfonyl **4**; and (d) partial gel, which had only part of the sample form effective self-supporting gel, observed from BA **9** (*para*-) *via* solvent switch method using 50% EtOH: H_2O at 1 %w/v.

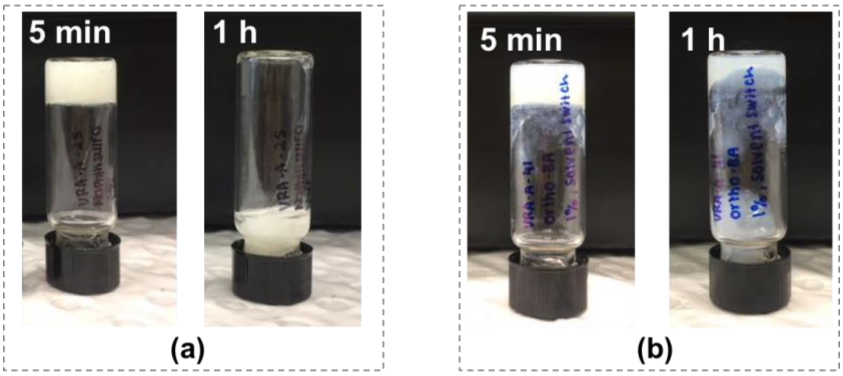

Figure S 4 (a) *N*-naphthalene sulfonyl **4** and (b) BA 7 (*ortho*-) formed opaque gels in the first 5 minutes which then collapsed after 1 hour *via* solvent switch method using 50% DMSO: H_2O.

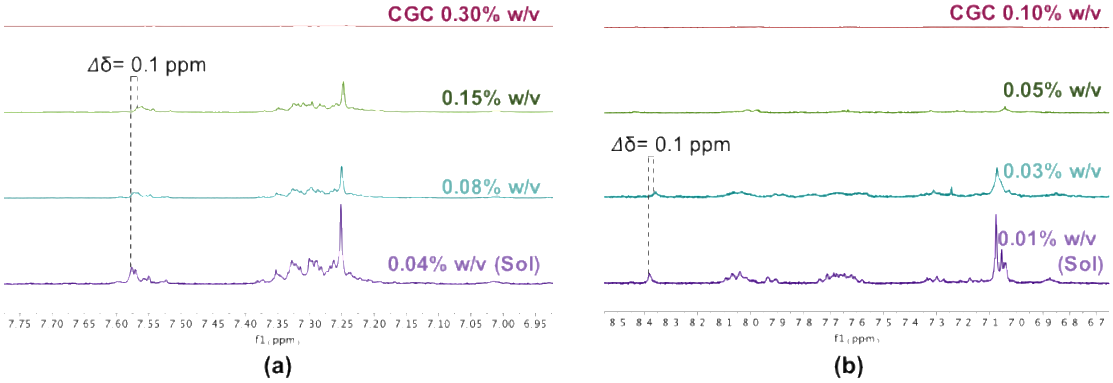

Figure S 5 ^{1}H NMR of (a) *N*-acetyl **1** and (b) *N*-benzoyl **2** showed up-field chemical shifts and peak broadening of aryl proton from aromatic cap as concentration of these short peptide was increased.

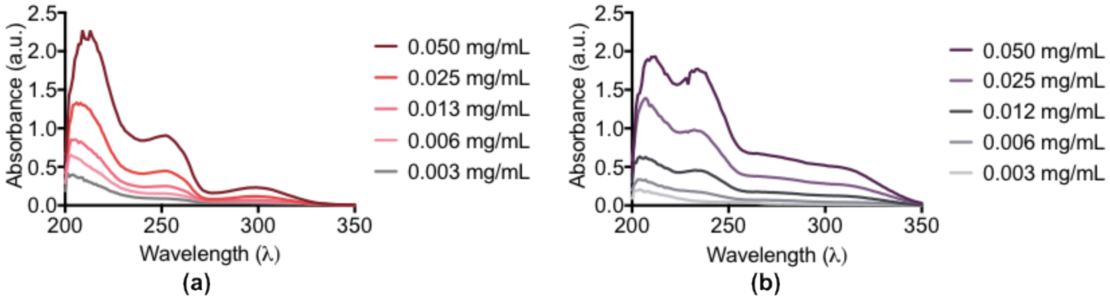

Figure S 6 Concentration dependent UV-Vis spectra of (a) *N*-acetyl **1** and (b)*N*-benzoyl **2** showed bathochromic shifts and enhancement of shoulder peaks which indicate involvement of aromatic capping group in self-assembly.

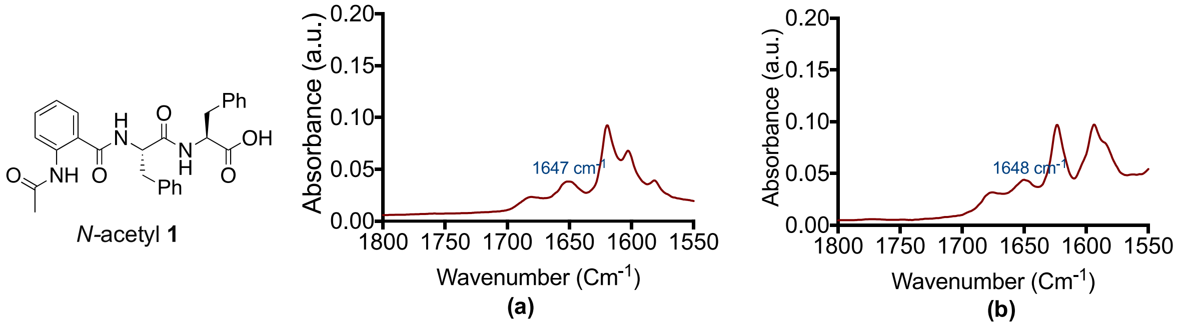

Figure S 7 ATR-FTIR spectra obtained from (a) D_2O gel and (b) xerogel of *N*-acetyl **1** exhibited disorder coil structure.

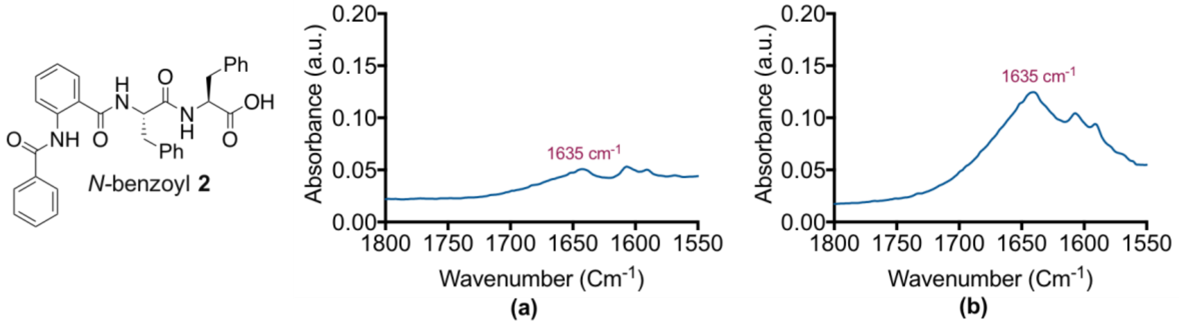

Figure S 8 ATR-FTIR spectra obtained from (a) D_2O gel and (b) xerogel of *N*-benzoyl **2** exhibited *β*-sheet structure.

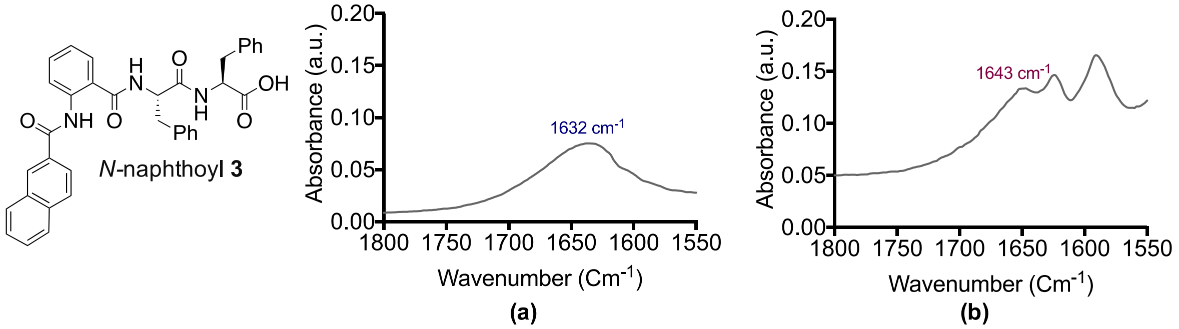

Figure S 9 ATR-FTIR spectra obtained from (a) D_2O gel and (b) xerogel of *N*-naphthoyl **3** exhibited *ß*-sheet structure.

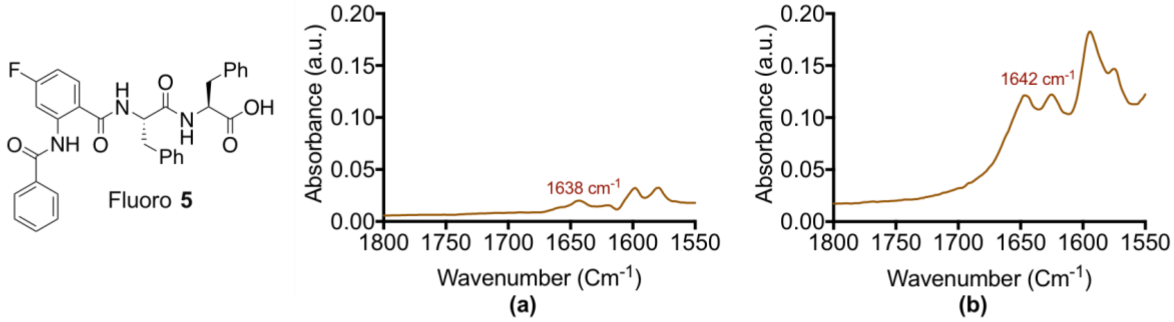

Figure S 10 ATR-FTIR spectra obtained from (a) D_2O gel and (b) xerogel of fluoro **5** exhibited *ß*-sheet structure.

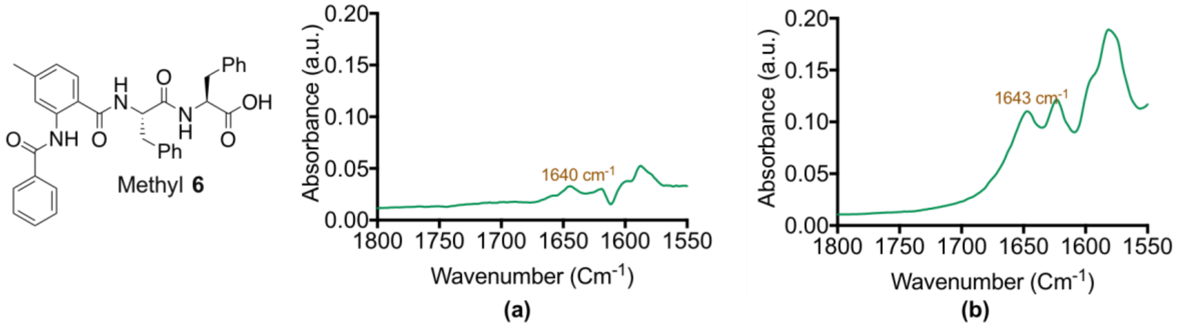

Figure S 11 ATR-FTIR spectra obtained from (a) D_2O gel and (b) xerogel of methyl **6** exhibited *ß*-sheet structure.

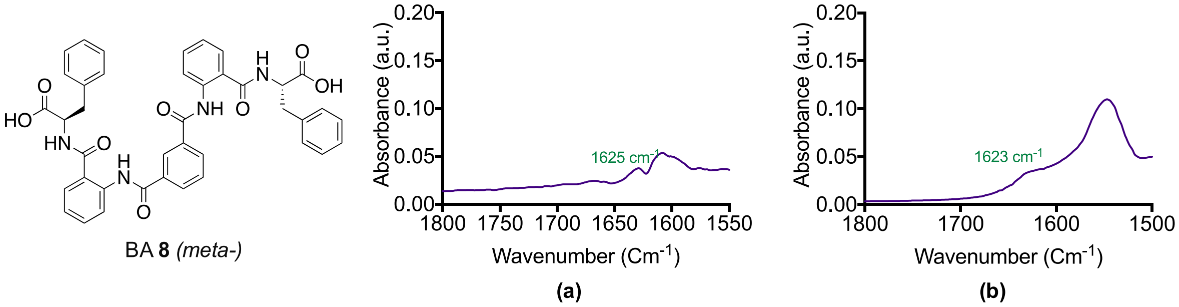

Figure S 12 ATR-FTIR spectra obtained from (a) D_2O gel and (b) xerogel of BA **8** (*meta*-) exhibited *ß*-sheet structure.

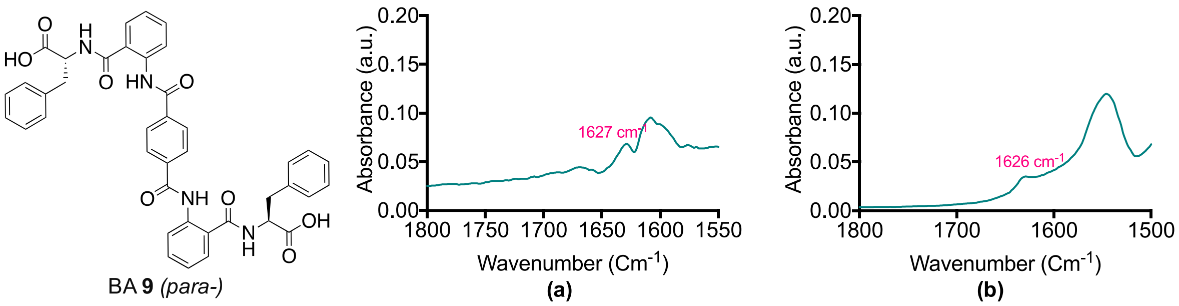

Figure S 13 ATR-FTIR spectra obtained from (a) D_2O gel and (b) xerogel of BA **9** (*para*-) exhibited *ß*-sheet structure.

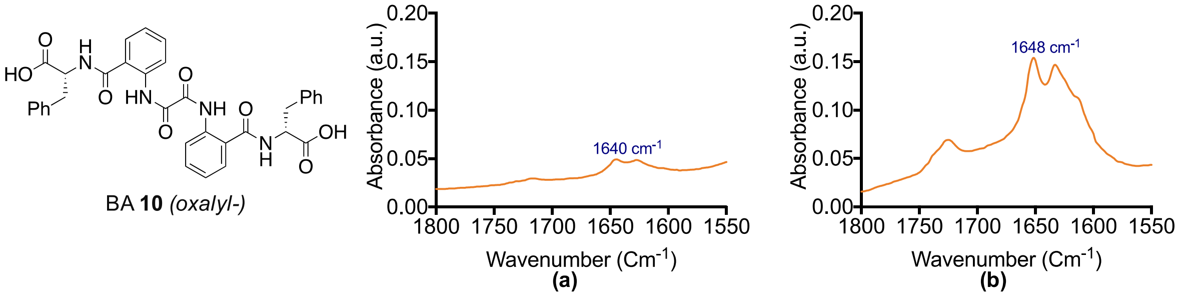

Figure S 14 ATR-FTIR spectra obtained from (a) D_2O gel and (b) xerogel of BA **10** (*oxalyl*-) exhibited disordered coil structure.

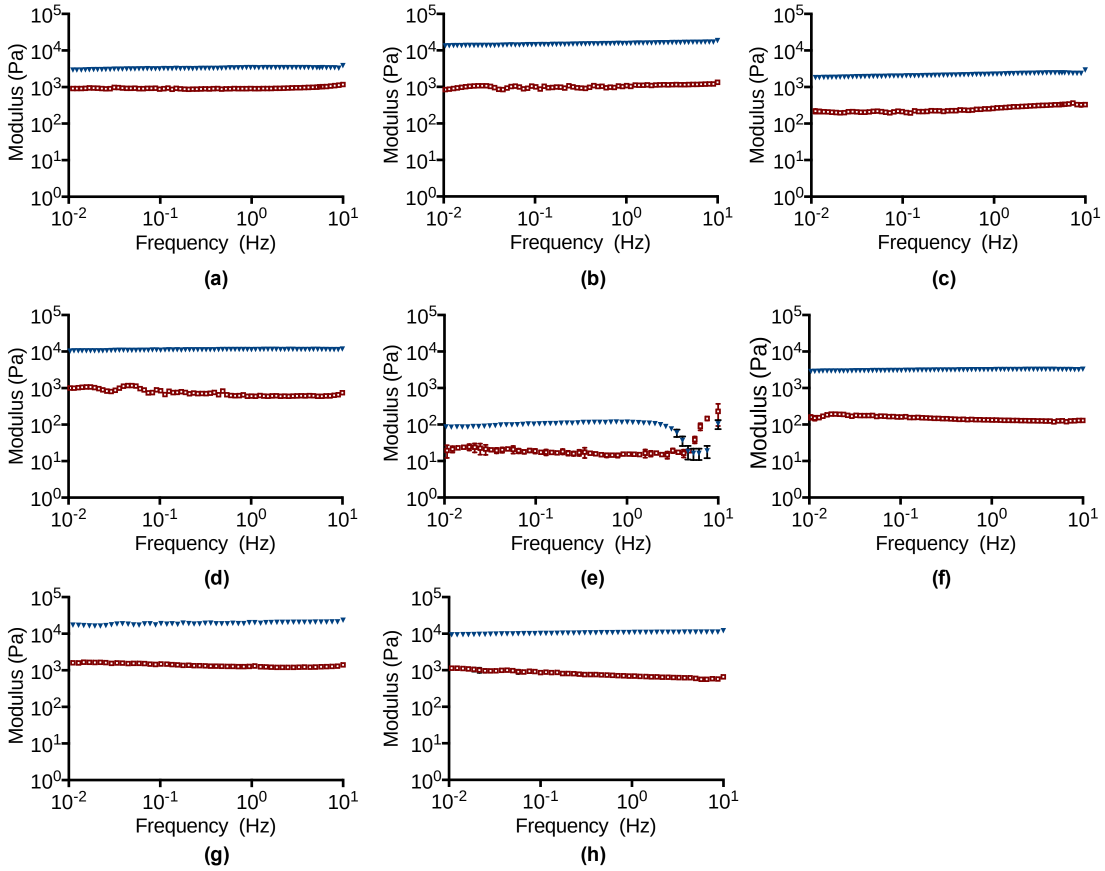

Figure S 15 Frequency sweep test (FST) of (a) *N*-acetyl **1**, (b) *N*-benzoyl **2**, (c) *N*-naphthoyl **3**, (d) fluoro **5**, (e) methyl **6**, (f) BA **8** (meta-), (g) BA **9** (para-), and (h) BA **10** (linked via oxalyl). Modulus storage (G′) is denoted with blue triangle, modulus loss (G″) denoted with red square. The graphs were representing three individual measurements and some of the error bars are on the order of graph point size.

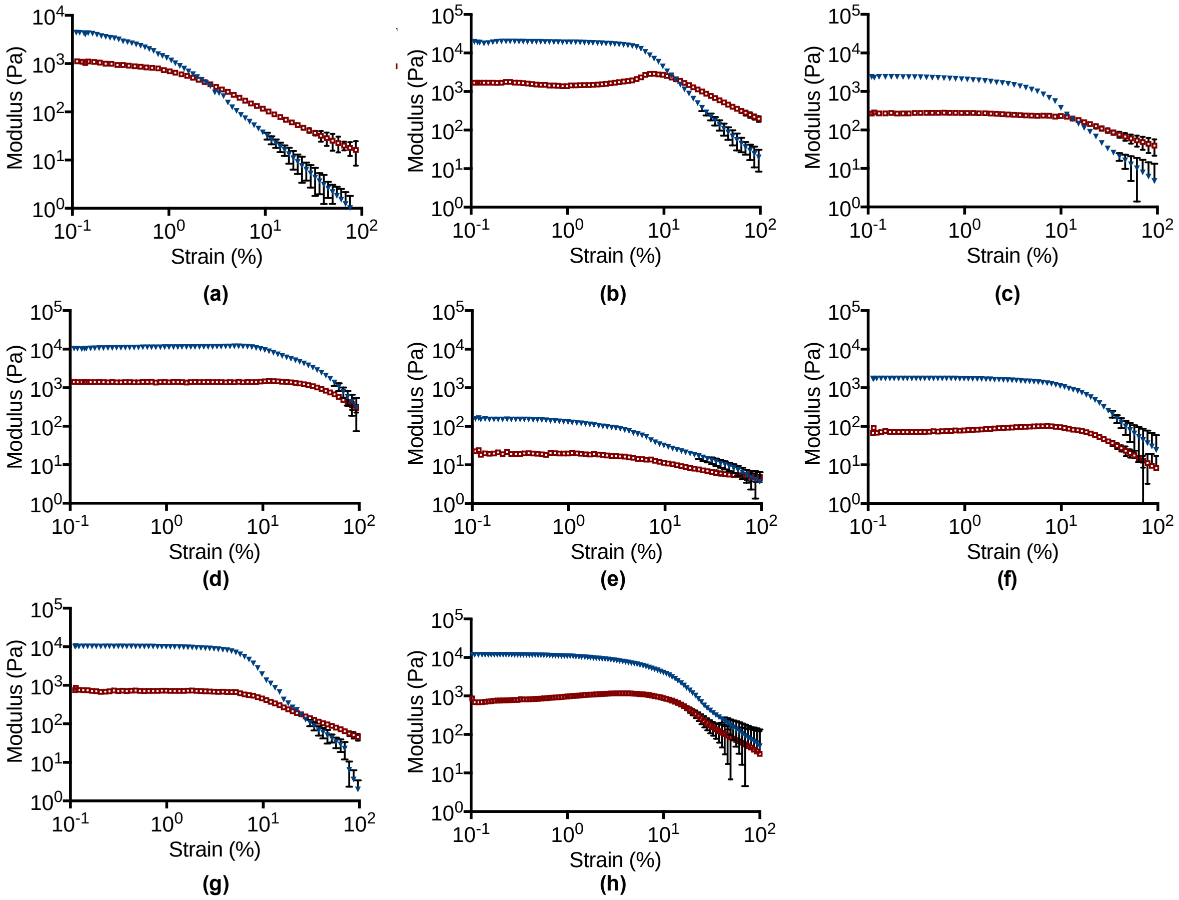

Figure S 16 Strain sweep test (SST) of (a) *N*-acetyl **1**, (b) *N*-benzoyl **2**, (c) *N*-naphthoyl **3**, (d) fluoro **5**, (e) methyl **6**, (f) BA **8** (meta-), (g) BA **9** (para-), and (h) BA **10** (linked *via* oxalyl) at 1 %w/v. Modulus storage (G′) is denoted with blue triangle, modulus loss (G″) denoted with red square. The graphs were representing three individual measurements and some of the error bars are on the order of graph point size.

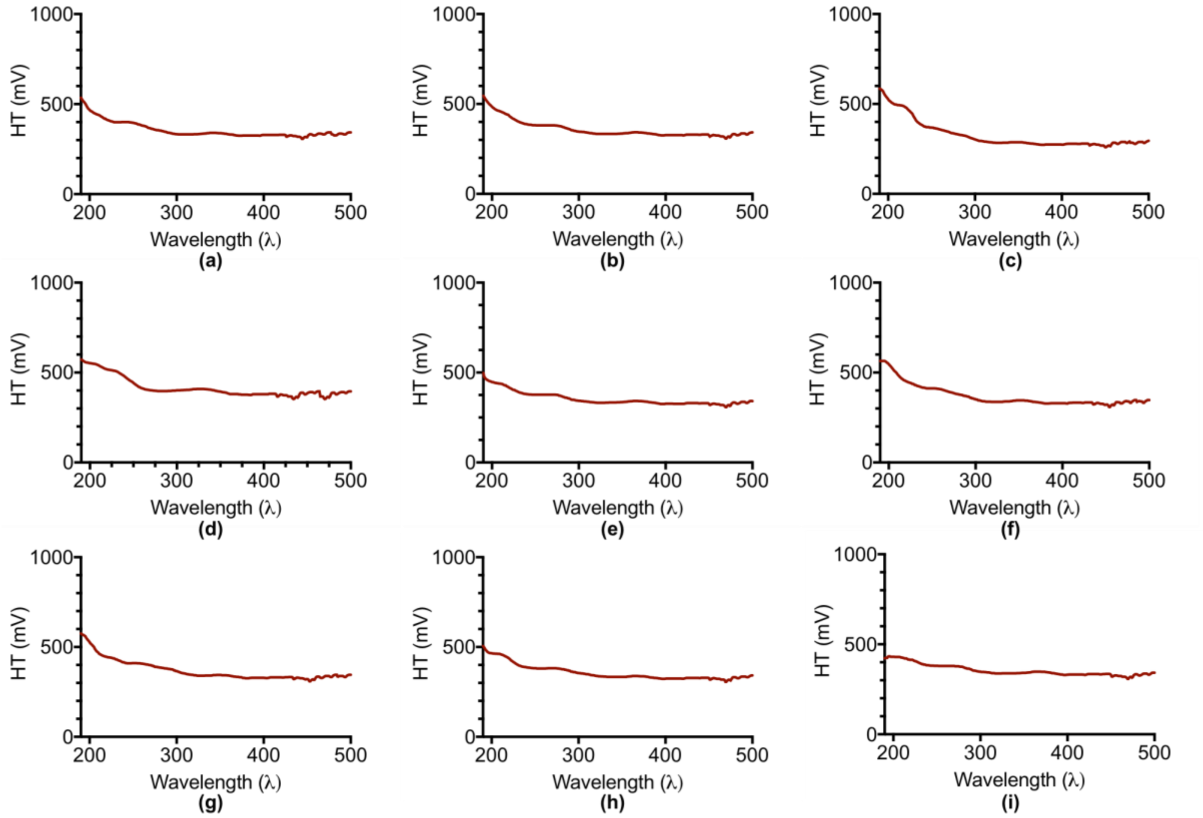

Figure S 17 High tension (HT) values obtained during CD spectroscopy measurement to determine the secondary structure of (a) *N*-acetyl **1**, (b) *N*-benzoyl **2**, (c) *N*-naphthoyl **3**, (d) fluoro **5**, (e) methyl **6**, (f) BA **8** (meta-), (g) BA **9** (para-), (h) BA **10** (linked *via* oxalyl) and (i) Mili-Q water as a blank were maintain to be bellow 600mV.

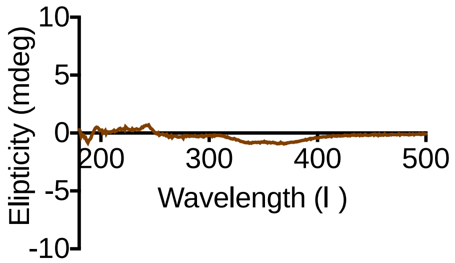

Figure S 18 CD spectrum of BA **9** (*para*-) at 0.08 %w/v, as a model compound, prior to gelation (in its monomer state) exhibited CD silent.

SYNTHESIS

All chemicals and solvents used were purchased from Chemimpex, Combi Blocks, or Sigma Aldrich and were used without any further purification.

General Procedure

General procedure for ring opening reaction of isatoic anhydride derivatives (GP1):

The commercially available L-phenylalanyl hydrochloride salt (1.0 equivalent) or methyl L-phenylalanyl-L-phenylalaninate hydrochloride salt (1.0 equivalent) was dissolved in Mili-Q water.

Potassium carbonate (K_2CO_3) (2.0 equivalent) was added to the reaction mixture and left to be stirred at room temperature for 20 minutes. The respective isatoic anhydride derivatives (in 5 mL of acetone) was added to the clear reaction mixture and stirred for 18h. After completion, the cloudy reaction mixture was then reduced under pressure to remove the acetone. The resulting white precipitate was filtered and dried to give compound **12a-c** or compound **13** as white solids in 65 – 85% yield.

General procedure for benzoylation and naphthoylation of compound 12a-c (GP2)

The Methyl (2-aminobenzoyl)-L-phenylalanyl-L-phenylalaninate or the substituted derivatives **12a-c** (1.0 equivalent) was suspended in pyridine (1 mL/ mmol) under nitrogen atmosphere and stirred for 10 minutes. After cooled down to 0 °C, the respective acyl chloride (i.e. benzoyl or naphthoyl chloride, 1.5 equivalent) was added dropwise. The reaction was then warmed to room temperature and stirred for 4-6 hours. After completion, the reaction mixture was poured into ice-water mixture, filtered, and dried. The resulting crude materials was purified using column chromatography to provide the ester intermediate as a pure white solid in 75- 92% yield.

General procedure for acylation reaction to obtain methyl ester protected bola amphiphile (GP3)

methyl (2-aminobenzoyl)-L-phenylalaninate **13** (1.0 equivalent) was dissolved in dichloromethane (DCM) under nitrogen atmosphere followed by addition of respective base (1.0 – 2.0 equivalent). The corresponding isomeric structure of benzene dicarbonyl dichloride (*ortho-, meta,* and *para-*) or oxalyl chloride (0.5 equivalent) was added dropwise to the reaction mixture and stirred for 18h. After completion, the resulting reaction mixture was diluted with 50 mL of DCM and subsequently washed with HCL (2M) and $NaHCO_3$. The organic phase was dried using sodium sulfate (Na_2SO_4) anhydrous and concentrated under reduced pressure. The crude materials were purified using column chromatography with hexane and ethyl acetate, as mobile phase, to obtain the methyl ester intermediates in 40-85% yield.

General procedure for hydrolysis (GP4):

The respective methyl ester intermediates were dissolved in a mixture of tetrahydrofuran, methanol, and water with ratio 10:5:2 (1mL/ mmol). Lithium hydroxide (LiOH) was added slowly to the clear

solution and stirred at room temperature for 18h. The reaction mixture was then diluted with Mili-Q water and washed with 3 x 15 mL diethyl ether. The aqueous phase was acidified (pH= 3-4) and was extracted with ethyl acetate 2 x 25 mL. The organic phase was dried over anhydrous sodium sulfate and concentrated under reduced pressure to give the final hydrogelators **1-11** in quantitative yields.

Starting Materials

Methyl L-phenylalanyl-L-phenylalaninate hydrochloride salt (FF·HCl)[1]

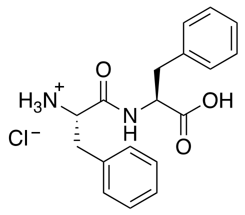

The commercially Boc-L-phenylanine (3.0 g, 0.01 mol) was coupled with L-phenylanine methyl ester hydrochloride salt (1.0 equivalent) using hydroxybenzotriazole (HOBT) (1.2 equivalent), N'-ethylcarbodiimide (EDC) (1.2 equivalent), and triethylamine (Et_3N) (2.0 equivalent) in DMF. The product was precipitated by addition of ice-water and the Boc-protecting group was removed using 4M HCl (2.0 equivalent) in dioxane to provide the pure product as a white solid (3.2 g, 78% yield). ^{1}H NMR (400 MHz, DMSO-d_6): 9.13 (1H, d, *J*= 7.5 Hz, *NH*), 8.16 (1H, s, *NH*), 7.24-7.32 (10H, m, *ArH*), 4.55 (1H, td, *J*= 10.0, 4.0 Hz, *CH*), 4.05 (1H, td, *J*= 8.4, 5.4 Hz, *CH*), 3.60 (3H, s, *$COOCH_3$*), 3.03-3.15 (2H, m, *CH_2*), 2.92-3.03 (2H, m, *CH_2*).

Ring-Opened Isatoic Anhydride Derivatives (12a-c and 13)

Methyl (2-aminobenzoyl)-L-phenylalanyl-L-phenylalaninate 12a

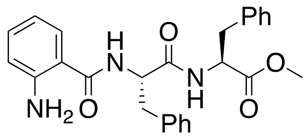

FF·HCl (5.0 gram, 13.7 mmol) was reacted with isatoic anhydride (1.0 equivalent) and K_2CO_3 (2.0 equivalent), according to **GP1.** After purification using column chromatography with hexane: ethyl acetate (60%), compound **12a** was obtained as a white solid in 85% yield (5.1 gram).

M.p.= 176.9 °C. IR Max: 3411, 3279, 3058, 2946, 1748, 1657, 1525, 1456, 1337, 1267, 1169, 1091, 1031, 919,804, 743, 697. ^{1}H NMR (DMSO-d_6, 400 MHz) 8.45 (1H, d, *J*= 8Hz, *ArH*), 8.18 (1H, d, 8.42, *NH*), 7.42 (1H, d, *J*= 8.0 Hz, *ArH*), 7.31 (2H, d, *J*= 7.1 Hz, *ArH*), 7.22 – 7.27 (6H, m, *ArH*), 7.09 – 7.22 (3H, m, *ArH*), 6.63 (1H, d, *J*= 8.3 Hz, *ArH*), 6.47 – 6.49 (1H, m, *ArH*), 6.28 (2H, s, *NH_2*), 4.64 – 4.70 (1H, m, *CH*), 4.49 – 4.54 (1H, m, *CH*), 3.59 (1H, s, *OCH_3*), 3.04 – 3.09 (2H, m, *CH_2*), 2.90 – 3.04 (2H, m, *CH_2*). ^{13}C NMR

(DMSO-d_6, 101 MHz) δ 171.81, 168.67, 149.63, 138.38, 137.03, 131.84, 129.13, 128.36, 128.25, 128.03, 126.57, 126.19, 116.20, 114.39, 114.07, 54.15, 53.65, 51.86, 36.81, 36.64. HR-MS (ESI): calcd for $C_{20}H_{27}N_3O_4$+H: 446.2080 found 446.2079.

Methyl (2-amino-5-fluorobenzoyl)-L-phenylalanyl-L-phenylalaninate 12b

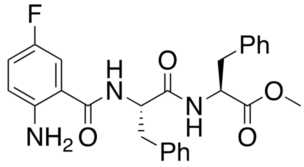

According to **GP1**, FF·HCl (1.0 gram, 2.8 mmol) was reacted with 5-fluoroisatoic anhydride (1.0 equivalent) to give compound **12b** as white solid (70% yield, 0.9 gram) after purification with hexane: ethylacetate (50%).

M.p. = 182.3 °C. IR Max: 3410, 3265, 3043, 2940, 1741, 1676, 1521, 1440, 1331, 1260, 1157, 1076, 1032, 867,804, 732, 678. ^{1}H NMR (300 MHz, DMSO-d_6) 8.52 (1H, d, *J* = 7.5 Hz, *NH*), 8.10 (1H, d, *J* = 8.4 Hz, *NH*), 7.38 – 7.12 (11H, m, *ArH*), 7.06 – 6.92 (1H, m, *ArH*), 6.63 (1H, d, *J* = 8.0 Hz, *ArH*), 6.00 (2H, s, *NH_2*), 4.75 – 4.58 (1H, m, *CH*), 4.51 (1H, td, *J* = 8.1; 6.0 Hz, *CH*), 3.59 (3H, s, *OCH_3*), 3.10 – 2.80 (4H, m, *CH_2*). ^{13}C NMR (DMSO-*d6*, 101 MHz) 171.82, 168.60, 147.21, 138.25, 137.00, 132.50, 129.11, 129.11, 128.24, 128.10, 126.60, 126.18, 122.82, 116.34, 114.19, 54.00, 53.61, 51.80, 36.85, 36.67. HR-MS (ESI): cald for $C_{26}H_{26}FN_3O_4$+H: 464.1986 found 464.1992.

Methyl (2-amino-5-methylbenzoyl)-L-phenylalanyl-L-phenylalaninate 12c

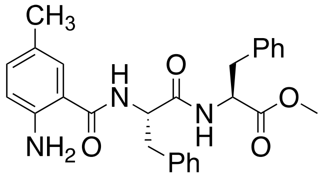

FF·HCl (1.0 gram, 2.8 mmol) was reacted with 5-methylisatoic anhydride (1.0 equivalent) according to **GP1**. The crude product was subjected to column chromatography using hexane: ethyl acetate (60%) to provide compound **12c** as a white solid in 82% yield (1.1 gram).

M.p.= 169.4 °C. IR Max: 3415, 3257, 3040, 2932, 1743, 1669, 1519, 1437, 1331, 1256, 1158, 1068, 1027, 837, 724, 683. ^{1}H NMR (300 MHz, DMSO-d_6) 8.48 (1H, d, *J* = 7.5 Hz, *NH*), 8.14 (1H, d, *J* = 8.4 Hz, *NH*), 7.41 – 7.12 (11H, m, *ArH*), 7.00 – 6.90 (1H, m, *ArH*), 6.56 (1H, d, *J* = 8.3 Hz, *ArH*), 6.06 (2H, s, *NH_2*), 4.76 – 4.60 (1H, m, *CH*), 4.51 (1H, td, *J* = 8.1; 6.0 Hz, *CH*), 3.59 (3H, s, *OCH_3*), 3.16 – 2.79 (4H, m, *CH_2*), 2.15 (3H, s, *CH_3*). ^{13}C NMR (DMSO-*d6*, 101 MHz) 171.79, 168.63, 147.31, 138.33, 137.04, 132.66,

129.14, 129.11, 128.25, 128.02, 126.56, 126.19, 122.76, 116.37, 114.15, 53.99, 53.66, 51.85, 36.86, 36.63, 19.99. HR-MS (ESI): cald for $C_{27}H_{29}N_3O_4$+H: 460.2236 found 460.2230.

Methyl (2-aminobenzoyl)-L-phenylalaninate 13[2,3]

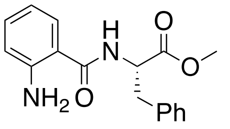

L-phenylalanyl hydrochloride salt (4.1 gram, 19.0 mmol) was used to ring open Isatoic anhydride (1.0 equivalent) in the presence of K_2CO_3 (2.0 equivalents) as a base according to **GP 1** to provide compound **13** as a white pure solid (3.7 gram, 65% yield). ^{1}H NMR (DMSO-*d6*, 400 MHz) 8.62 (1H, d, *J*= 7.6 Hz, *NH*), 7.56 (1H, d, *J*= 7.8 Hz *ArH*), 7.36 – 7.41 (4H, m, *ArH*), 7.28 – 7.31 (1H, m, *ArH*), 7.21 -7.25 (1H, m, *ArH*), 6.76 (1H, d, *J*= 8.4 Hz, *ArH*), 6.59 (1H, t, *J*= 7.8 Hz, *ArH*), 6.42 (2H, bs, *NH$_2$*), 4.67 – 4.73 (1H, m, *CH*), 3.73 (3H, s, *OCH$_3$*), 3.15 – 3.26 (2H, m, *CH$_2$*).

Methyl Ester Protected Hydrogelators

Methyl (2-acetamidobenzoyl)-L-phenylalanyl-L-phenylalaninate

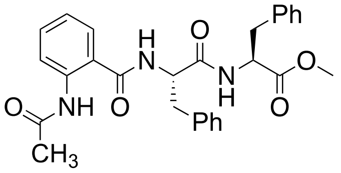

Methyl (2-aminobenzoyl)-L-phenylalanyl-L-phenylalaninate **12a** (308 mg, 0.7 mmol) was suspended in anhydrous dichloromethane (6mL) under nitrogen atmosphere. Et_3N (2.0 equivalent) was added to the white suspension and stirred at room temperature for 10 minutes. Acetic acid anhydride (2.0 equivalents) was added dropwise to the colorless solution. The reaction mixture was then heated at 40 °C for 18h. After completion, the reaction mixture was diluted with 30 mL of DCM and washed with $NaHCO_3$ and brine to remove the excess acetic anhydride. The resulting organic phase was dried over Na_2SO_4 and concentrated under reduced pressure. The crude material was purified using column chromatography with hexane: ethyl acetate (50%) to give a pure white solid in 85% yield (290 mg).

M.p.= 186.0 °C. IR Max: 3281, 2930, 1738, 1696, 1626, 1594, 1512, 1439, 1374, 1278, 1215, 1111, 1030, 869, 746. ^{1}H NMR (DMSO-*d6*, 400 MHz) 10.66 (1H, m s, *NH*), 8.76 (1H, d, *J*= 8.5 Hz, *NH*), 8.61 (1H, d, *J*= 7.8 Hz, *NH*), 8.22 (1H, d, *J*= 8.3 Hz, *ArH*), 7.52 (1H, dd, *J*= 7.8; 1.3 Hz, *ArH*), 7.42 7.46 (1H, m, *ArH*), 7.32 (2H, d, *J*= 7.2 Hz, *ArH*), 7.22 – 7.28 (6H, m, *ArH*), 7.16 – 7.21 (2H, m, *ArH*), 7.10 (1H, td, *J*= 7.8; 1.3 Hz, *ArH*), 4.70 – 4.7 (1H, m, *CH*), 4.52 – 4.57 (1H, m, *CH*), 3.60 (3H, s, *OCH$_3$*), 3.07 – 3.14 (2H, m, *CH$_2$*), 2.88 – 3.05 (2H, m, CH_2), 1.99 (3H, s, CH_3). ^{13}C NMR (DMSO-*d6*, 101 MHz) 1717.76, 171.30,

168.21, 168.08, 138.22, 138.11, 137.07, 131.62, 129.14, 129. 09, 128.23, 128.15, 128.04, 126.57, 126.29, 122.56, 121.92, 120.50, 54.41, 53.81, 51.89, 36.75, 36.46, 24.57. HR-MS (ESI): calcd for $C_{28}H_{29}N_3O_5$+Na: 510.2005 found 510.1997.

Methyl (2-benzamidobenzoyl)-L-phenylalanyl-L-phenylalaninate

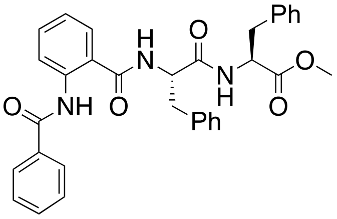

According to **GP2**, methyl (2-aminobenzoyl)-L-phenylalanyl-L-phenylalaninate **12a** (503 mg, 1.1 mmol) was reacted with benzoyl chloride (1.5 equivalent) in room temperature for 4 hours. The crude mixture was purified using column chromatography with hexane: ethyl acetate (50%) to obtain pure white solid in 91% yield (593 mg).

M.p.= 215.4-216.0 °C. IR Max: 3294, 2925, 2648, 2117, 1737, 1656, 1590, 1519, 1440, 1380, 1286, 1240, 1160, 1027, 918, 749, 697. ^{1}H NMR (DMSO-*d6*, 400 MHz) 12.08 (1H, s, *NH*), 8.98 (1H, d, J= 9.14 Hz, *NH*), 8.59-8.63 (2H, m, *ArH*), 7.81 (2H, d, *J*= 7.50Hz, *ArH*), 7.75 (1H, d, *J*= 7.50, *ArH*), 7.59 – 7.61 (1H, m, *ArH*), 7.51 – 7.55 (1H, m, *ArH*), 7.34 (2H, d, *J*= 7.55, *ArH*), 7.14 – 7.22 (7H, m, *ArH*), 7.07 – 7.12 (2H, m, *ArH*), 4.80 -4.86 (1H, m, *CH*), 4.52 – 4.55 (1H, m, *CH*), 3.57 (3H, s, *OCH_3*), 3.05 – 3.12 (2H, m, *CH_2*), 2.92 – 3.00 (2H, m, *CH_2*). ^{13}C NMR (DMSO-*d6*, 101 MHz) 171.67, 171.02, 168.54, 164. 32, 139.17, 138.06, 137, 134.45, 132.38, 132.04, 129.12, 129.03, 128.85, 128.50, 128. 15, 127.98, 126.89, 126.49, 126.26, 122.69, 120.24, 119.97, 54.45, 53.61, 51.84, 36.84, 36.44. HR-MS (ESI): calcd for $C_{33}H_{31}N_3O_5$+Na: 572.2161 found 572.2153

Methyl (2-(2-naphthamido)benzoyl)-L-phenylalanyl-L-phenylalaninate

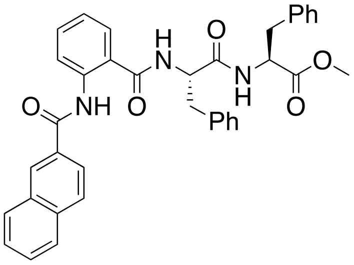

Methyl (2-aminobenzoyl)-L-phenylalanyl-L-phenylalaninate **12a** (740 mg, 1 .6 mmol) was reacted with naphthoyl chloride (1.5 equivalents), according to **GP 2,** for 6 hours. The crude mixture was purified using hexane: ethyl acetate (30%) to obtain pure white fluffy solid in 80% yield (797 mg).

M.p. = 218.9-219.3 °C. IR Max: 3282, 2928, 2322, 1739, 1560, 1519, 1446, 1280, 1248, 1032, 914, 749, 699. ^{1}H NMR (DMSO-d6, 400 MHz) 12.21 (1H, bs, NH), 9.01 (1H, d, *J*= 8.2 Hz, *NH*), 8.62 (2H, t, *J*= 6.8,

ArH) 8.44 (1H, bs, *NH*), 8.00 – 8.09 (3H, m, *ArH*), 7.86 (1H, dd, *J*= 8.3; 1.5 *ArH*), 7.77 (1H, dd, *J*= 8.3; 1.5 Hz, *ArH*), 7.55 – 7.68 (3H, m, *ArH*), 7.35 (2H, d, *J*= 7.40 Hz, *ArH*), 7.00 – 7.23 (10H, m, *ArH*), 4.81 - 4.89 (1H, m, *CH*), 4.50-4.57 (1H, m, *CH*), 3.54 (3H, s, OCH_3), 3.04 – 3.14 (2H, m, CH_2), 2.92 – 3.04 (2H, m, CH_2). ^{13}C NMR (DMSO-*d6*, 101 MHz) 171.69, 171.04, 168.54, 164.46, 139.16, 138.08, 136.99, 134.41, 132.38, 132.20, 131.87, 129.13, 129.08, 128.99, 128.58, 128.10, 127.99, 127.77, 127.70, 127.05, 126.41, 126.25, 123.24, 122.79, 120.25, 54.46, 53.62, 51.83, 36.86, 36.45. HR-MS (ESI): calcd for $C_{37}H_{33}N_3O_5$+Na: 622.2318 found 622.2310.

Methyl (2-(naphthalene-1-sulfonamido)benzoyl)-L-phenylalanyl-L-phenylalaninate

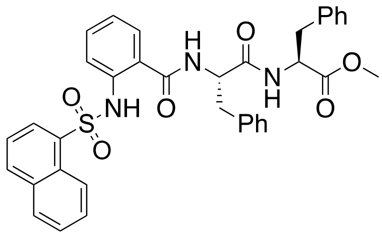

Methyl (2-aminobenzoyl)-L-phenylalanyl-L-phenylalaninate **12a** (420 mg, 0.94 mmol) was suspended in dry dichloromethane under nitrogen atmosphere. Dimethyl amino pyridine (DMAP) (1.0 equivalent) was added to the reaction mixture and cooled down to 0 °C. After 10 minutes, naphthalene sulfonyl chloride (1.0 equivalent) was added to the reaction mixture. The yellowish white reaction mixture was warmed to room temperature and stirred for 18 hours. After completion, the reaction mixture was diluted with 30 mL of DCM and washed with dilute HCl. The organic phase was dried over sodium sulfate, filtered, and concentrated under reduced pressure. The yellow crude was purified by column chromatography using hexane: ethyl acetate (50%) to provide pale yellow solid in 75% yield (450 mg).

M.p.= 83 °C. IR Max: 3277, 3028.9, 2950, 2311, 2111, 1737, 1632, 1509, 1449, 1331, 1263, 1211, 1159, 1028, 980, 928, 860, 803, 746, 698. ^{1}H NMR (DMSO-*d6*, 400 MHz). 12.10 (1H, bs, *NH*), 8.82 (1H, d, *J*= 8.5, *NH*), 8.68 (1H, d, *J*= 7.8 Hz, *NH*), 8.41 (1H, d, *J*= 7.8 Hz, *ArH*), 8.28 (1H, dd, *J*= 6.9; 1.1 Hz, *ArH*), 8.21 (1H, d, *J*= 8.1Hz, *ArH*), 8.02 (1H, dd, *J*= 8.1; 1.1 Hz, *ArH*), 7.62 – 7.67 (2H, m, *ArH*), 7.55 -7.60 (1H, m, *ArH*), 7.48 – 7.52 (1H, m, *ArH*), 7.22 - 7.34 (11H, m, *ArH*), 7.13 – 7.20 (3H, m, *ArH*), 6.96 – 7.00 (1H, m, *ArH*), 4.72 – 4.78 (1H, m, *CH*), 4.54 – 4.60 (1H, m, *CH*), 3.62 (3H, s, OCH_3), 2.88 – 3.13 (4H, m, CH_2). ^{13}C NMR (DMSO-*d6*, 101 MHz) 172.76, 170.91, 168.04, 137.99, 137.46, 134.89, 133.72, 132.74, 130.32, 129.20, 129.16, 129.08, 128.80, 128.44, 128.21, 128.08, 127.08, 127.03, 126.51, 126.32, 124.41,

123.37, 122.25, 118.09, 116.99, 54.58, 53.65, 51.98 36.76, 36.63. HR-MS (ESI): calcd $C_{36}H_{33}N_3O_6S$+Na: 658.1988 found 658.1977.

Methyl (2-benzamido-5-fluorobenzoyl)-L-phenylalanyl-L-phenylalaninate

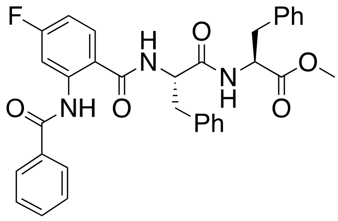

Methyl (2-amino-5-fluorobenzoyl)-L-phenylalanyl-L-phenylalaninate **12b** (650 mg, 1.4 mmol) was reacted with benzoyl chloride (1.5 equivalents) according to **GP2**. The yellowish white crude mixture was purified by column chromatography with hexane : ethyl acetate (40%) to give a white fluffy solid in 80% yield (635 mg).

M.p. = 189.6 °C. IR Max: 3296, 3061, 2949, 2097, 1737, 1656, 1960, 1522, 1412, 1294, 1237, 1091, 1038, 948, 897, 833, 692. ^{1}H NMR (DMSO-d6, 400 MHz) 11.87 (1H, s, *NH*), 9.05 (1H, d, J= 8.41 Hz, *NH*), 8.64 (1H, d, *J*= 8.41, *NH*) 8.57 (1H, dd, *J*= 9.46; 5.26 Hz, *ArH*), 7.80 (2H, dd, *J*= 7.12, 1.36Hz, *ArH*), 7.58 – 7.62 (2H, m, *ArH*), 7.51 – 7.55 (2H, m, *ArH*), 7.43 – 7.45 (1H, m, *ArH*), 7.32 (2H, d, *J*= 7.14*, ArH*), 7.14 – 7.22 (6H, m, *ArH*), 7.06 – 7.12 (2H, m, *ArH*), 4.79 -4.85 (1H, m, *CH*), 4.51-4.56 (1H, m, *CH*), 3.57 (3H, s, *OCH_3*), 3.05 – 3.14 (2H, m, *CH_2*), 2.88 – 3.14 (2H, m, *CH_2*). ^{13}C NMR (DMSO-*d6*, 101 MHz) 171.68, 170.84, 167.28, 164.32, 158.08, 155.69, 137.96, 137.07, 135.64, 134.28, 132.12, 129.24, 129.11, 129.05, 128.87, 128.16, 128.01, 126.92, 126.49, 126.31, 122.30, 122.22, 121.79, 121.73, 119.18, 118.96, 115.20, 114.95, 54.46, 53.67, 51.88, 36.87, 36.41. HR-MS (ESI): calcd $C_{33}H_{30}FN_3O_5$+Na: 590.2067 found 590.2060.

methyl (2-benzamido-5-methylbenzoyl)-L-phenylalanyl-L-phenylalaninate

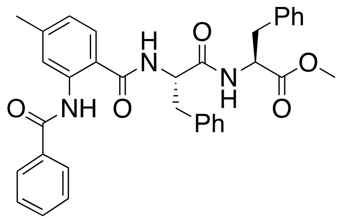

Methyl (2-amino-5-methylbenzoyl)-L-phenylalanyl-L-phenylalaninate **12c** (530 mg, 1.2 mmol) was reacted with benzoyl chloride (1.5 equivalents) according to **GP2**. The crude product was purified using hexane: ethyl acetate (30%) to obtain pure white powder in 92% yield (595 mg).

M.p.= 207.1 °C. IR Max: 3497, 3304, 2953, 2853, 2184, 1738, 1660, 1587, 1522, 1455, 1387, 1238, 1108, 1028, 917, 830, 678. ^{1}H NMR (DMSO-*d6*, 400 MHz) 11.93 (1H, s, *NH*), 8.90 (1H, d, *J= 8.7* Hz, *NH*),

8.61 (1H, d, *J*= 7.6, *NH*), 8.46 (1H, d, *J*= 8.7 Hz, *ArH*), 7.79 2H (2H, dd, J= 7.1; 1.4 Hz, *ArH*), 7.57 – 7.63 (2H, m, *ArH*), 7.48 – 7.54 (2H, m, *ArH*), 7.32 – 7.38 (3H, m, *ArH*), 7.14 – 7.22 (6H, m, *ArH*), 7.04 – 7.12 (2H, m, *ArH*), 4.81 – 4.86 (1H, m, *CH*), 4.51 – 4.57 (1H, m, *CH*), 3.57 (3H, s, OCH_3), 2.91 – 2.98 (2H, m, CH_2), 3.00 – 3.12 (2H, m, CH_2), 2.32 (3H, s, CH_3). ^{13}C NMR (DMSO-*d6*, 101 MHz) 171.69, 171.07, 168.52, 164.12, 138.03, 137.04, 136.74, 134.56, 132.75, 131.94, 131.74, 129.25, 129.15, 129.04, 128.82, 128.79, 128.54, 128.16, 127.97, 126.86, 126.50, 126.27, 120.03, 120.00,54.91, 54.30, 53.65, 51.86, 36.94, 36.45, 20.40. HR-MS (ESI): calcd $C_{34}H_{33}N_3O_5$+Na: 586.2318 found 586.2310

methyl (2-(2-((2-(((*R*)-1-methoxy-1-oxo-3-phenylpropan-2-yl)carbamoyl)phenyl)carbamoyl)-benzamido)benzoyl)-L-phenylalaninate

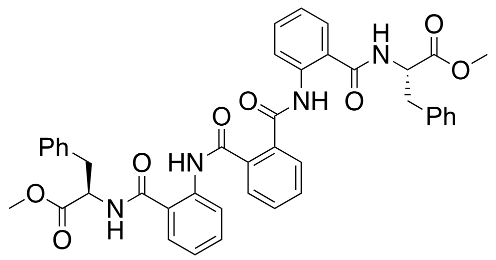

According to **GP3**, methyl (2-aminobenzoyl)-L-phenylalaninate **13** (400 mg, 1.3 mmol) was reacted with phthaloyl chloride (0.5 equivalent) to give brownish white solid. The crude mixture was then purified by column chromatography using hexane: ethyl acetate (60%) to obtain pure compound as a white solid in 40% yield (195 mg).

IR Max= 3342, 3301, 2950, 1748, 1683, 1631, 1600, 1522, 1442, 1361, 1302, 1213, 1175, 982, 932, 756, 702. M.p.= 180.1 – 180.4 °C. ^{1}H NMR (DMSO-*d6*, 400 MHz) 11.56 (2H, s, *NH*), 9.19 (2H, d, *J*= 8.0 Hz, *NH*), 8.43 (2H, d, *J*= 8.4 Hz, *ArH*), 7.73 – 7.76 (2H, m, *ArH*), 7.65 – 7.70 (2H, m, *ArH*), 7.49 – 7.61 (4H, m, *ArH*), 7.27 (4H, s, *ArH*), 7.25 (4H, d, *J*= 7.3 Hz, *ArH*), 7.18 – 7.20 (5H, m, *ArH*), 4.63 – 4.70 (2H, m, *CH*), 3.61 (6H, s, *OCH_3*), 3.06 – 3.26 (4H, m, CH_2). ^{13}C NMR (DMSO-*d6*, 101 MHz)170.59, 168.43, 165.87, 139.00, 137.45, 136.43, 132.40, 130.88, 129.05, 128.24, 128.23, 127.27, 126.55, 122.98, 120.45, 120.00, 54.04, 52.05, 36.09. HR-MS (ESI): calcd $C_{42}H_{38}N_4O_8$+Na: 749.2587 found 749.2581.

Methyl (2-(3-((2-(((*R*)-1-methoxy-1-oxo-3-phenylpropan-2-yl)carbamoyl)phenyl)-carbamoyl)-benzamido)benzoyl)-L-phenylalaninate.

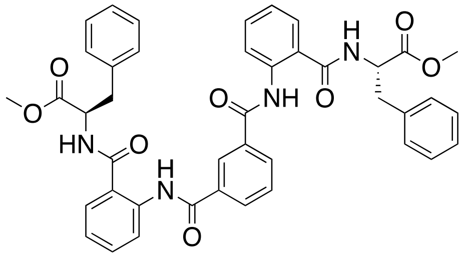

Compound **13** (600 mg, 1.0 equivalent) was reacted with isophthtaloyl chloride (0.5 equivalent) in the presence of pyridine (2.0 equivalents) and DMAP (0.05 equivalent) according to **GP3**. The brownish-white crude mixture was purified with hexane: ethylacetate (60%) to obtain fluffy white solid in 40% yield (300 mg).

IR Max= 3346, 3304, 2954, 1744, 1681, 1633, 1600, 1520, 1441, 1359, 1300, 1214, 1170, 983, 936, 755, 699. M.p.= 193.8 – 194.2 °C. ^{1}H NMR (DMSO-*d6*, 400 MHz) 12.06 (2H, s, *NH*), 9.26 (2H, d, *J*= 8.0 Hz, *NH*), 8.57 – 8.60 (2H, m, *ArH*), 8.43 (1H, S, *ArH*), 8.01 (2H, dd, *J*= 7.8; 1.3, *ArH*), 7.76 – 7.82 (3H, m, *ArH*), 7.57 – 7.63 (2H, m, *ArH*), 7.19 – 7.31 (10H, m, *ArH*), 7.08 – 7.13 (2H, m, *ArH*), 4.76 – 4.84 (2H, m, *CH*), 3.64 (6H, s, OCH_3), 3.06 – 3.26 (4H, m, CH_2). ^{13}C NMR (DMSO-*d6*, 101 MHz)171.62, 168.70, 163.63, 138.83, 137.42, 135.20, 132.53, 129.75, 129.68, 129.06, 128.39, 128.17, 126.49, 123.18, 120.61, 120.39, 54.04, 52.09, 36.15. HR-MS (ESI): calcd $C_{42}H_{38}N_4O_8$+Na: 749.2587 found 749.2577.

Methyl (2-(4-((2-(((*R*)-1-methoxy-1-oxo-3-phenylpropan-2-yl)carbamoyl)phenyl)-carbamoyl)-benzamido)benzoyl)-L-phenylalaninate

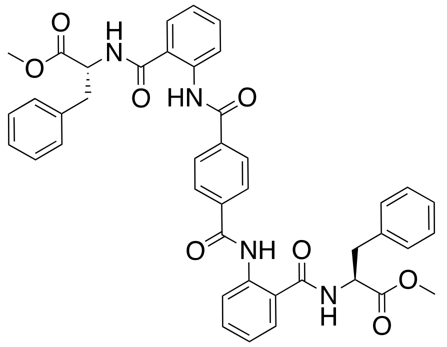

Compound **13** (900 mg, 3.0 mmol) was reacted with terephthaloy chloride (0.5 equivalent) in the presence of DIPEA (4.0 equivalents) as a base according to **GP3.** The crude mixture was purified by trituration with hexane to give pure white solid in 85% yield (930 mg).

M.p.= 213.0 – 213.2 °C. IR Max: 3316, 2951, 2659, 1946, 1752, 1676, 1624, 1587,1516, 1438, 1296, 1217, 1171, 1122, 1025, 891, 749, 696. ^{1}H NMR (DMSO-*d6*, 400 MHz) 12.04 (2H, s, *NH*), 9.26 (2H, d, *J*= 8.0 Hz, *NH*), 8.58 (2H, d, *J*= 8.8 Hz, *ArH*), 7.96 (4H, s, *ArH*), 7.78 (2H, d, *J*= 8.2 Hz, *ArH*), 7.60 (2H, t, *J*= 8.2 Hz, *ArH*), 7.31 (4H, d, *J*= 8.0 Hz, *ArH*), 7.22 – 7.27 (6H, m, *ArH*), &.11 (2H, t, *J*= 7.3 Hz, *ArH*), 4.80 – 4.86 (2H, m, *CH*), 3.66 (6H, s, OCH_3), 3.06 – 3. 26 (4H, m, CH_2). ^{13}C NMR (DMSO-*d6*, 101 MHz) 171.65, 168.72, 163.54, 138.80, 137.46, 137.38, 132.60, 129.11, 128.41, 128.20, 127.54, 126.54, 123.25, 120.46, 120.32, 54.08, 52.14, 36.20. HR-MS (ESI): calcd $C_{42}H_{38}N_4O_8$+Na: 749.2587 found 749.2580.

Methyl (2-(2-((2-(((*R*)-1-methoxy-1-oxo-3-phenylpropan-2-yl)carbamoyl)phenyl)amino)-2-oxoacetamido)benzoyl)-L-phenylalaninate

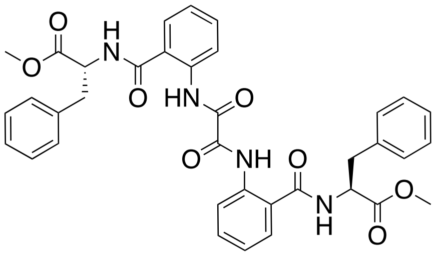

According to **GP3**, compound **13** (500 mg, 1.7 mmol) was reacted with oxaly chloride (0.5 equivalents) in the presence of pyridine (1.0 equivalents) as a base. After 16 hours, white precipitate was observed. The reaction mixture was diluted with 20mL of DCM, filtered, and dried. The resulting white solid (818 mg, 75% yield) was identified as pure product.

M.p.= 285.8 °C. IR Max 3288, 2949, 1731, 1693, 1582, 1512, 1447, 1221, 1298, 1232, 1178, 878, 755, 695. ^{1}H NMR (DMSO-*d6*, 400 MHz) 12.48 (2H, s, *NH*), 9.19 (2H, d, *J*= 7.8 *Hz*, *NH*), 8.53 (2H, d, *J*= 8.2 *Hz*, *ArH*), 7.78 (2H, d, *J*= 7.8 *Hz*, *ArH*), 7.60 (2H, t, *J*= 7.8 *Hz*, *ArH*), 7.24 – 7.32 (10H, m, *ArH*), 7.15 (2H, t, *J*= 7.3*Hz*, *ArH*), 4.72 -4.78 (2H, m, *CH*), 3.67 (6H, s, OCH_3), 3.08 – 3.24 (4H, m, CH_2). ^{13}C NMR (DMSO-*d6*, 101 MHz) 171.79, 167.85, 157.73, 137.47, 137.28, 132.55, 129.06, 128.49, 128.26, 126.51, 124.00, 120.79, 120.13, 54.05, 52.13, 36.10. HR-MS (ESI): calcd $C_{36}H_{34}N_4O_8$+Na: 673.2274 found 673.2279.

Methyl (2-(3-((2-(hydroxy(((*R*)-1-methoxy-1-oxo-3-phenylpropan-2-yl)amino)methyl)-phenyl)-carbamoyl)-5-((2-(((*R*)-1-methoxy-1-oxo-3-phenylpropan-2yl)carbamoyl)phenyl)carbamoyl)-benzamido)benzoyl)-L-phenylalaninate

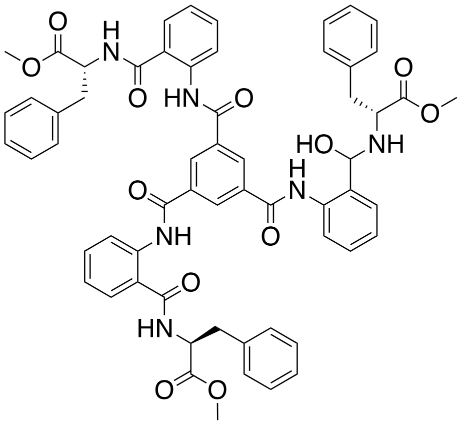

Compound **13** (1 gram, 3.4 mmol) was dissolved in anhydrous DCM under nitrogen atmosphere. DIPEA (3.0 equivalents) was added to the clear solution followed by trimesoyl chloride (0.3 equivalent). The reaction mixture was stirred in room temperature for 18h. The resulting white precipitate was collected by filtration and was identified as pure product in 50% yield (520 mg).

IR Max: 3315, 2661, 2493, 2111, 1745, 1641, 1589, 1511, 1444, 1302, 1217, 1130, 1022, 759, 699. M.p. 223.4 – 223.6 °C. ^{1}H NMR (DMSO-*d6*, 400 MHz) 12.05 (3H, s, *NH*), 9.22 (3H, d, *J*= 7.8 Hz, *NH*), 8.51 8.53

(6H, m, *ArH*), 7.77 (3H, d, *J*= 7.9 Hz, *ArH*), 7.60 -7.64 (3H, m, *ArH*), 7.25 – 7.30 (9H, m, *ArH*), 7.15 – 7.19 (6H, m, *ArH*), 7.06 – 7.08 (3H, m, *ArH*), 4.74 – 4.79 (3H, m, *CH*), 3.58 (9H, s, OCH_3), 3.04 – 3.20 (6H, m, CH_2). ^{13}C NMR (DMSO-*d6*, 101 MHz) 171.62, 168.54, 163.18, 138.44, 137.35, 136.04, 132.45, 129.04, 128.61, 128.44, 128.14, 126.44, 123.58, 121.20, 121.12, 53.99, 52.01, 36.17. HR-MS (ESI): calcd $C_{60}H_{54}N_6O_{12}$+Na: 1073.3697 found 1073.3682.

Final Hydrogelators 1-11

(2-Acetamidobenzoyl)-L-phenylalanyl-L-phenylalanine 1

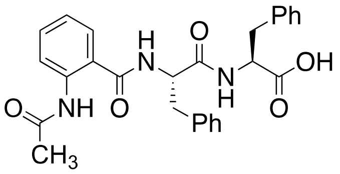

According to **GP 4**, methyl (2-aminobenzoyl)-L-phenylalanyl-L-phenylalaninate (250 mg, 0.5 mmol) reacted with LiOH (2.0 equivalents) for 18h to obtained hydrogelator **1** as a white powder in quantitative yields (240 mg).

M.p.= 212.7 – 213.2 °C. IR Max: 3286, 2934, 2240, 2143, 1738, 1627, 1515, 1441, 1376, 11216, 1108, 1029, 745, 698. ^{1}H NMR (DMSO-*d6*, 400 MHz) 12.80 (1H, bs, *OH*), 10.65 (1H, s, *NH*), 8.76 (1H, d, *J*= 8.4 Hz, *NH*), 8.43 (1H, d, *J*= 7.7 Hz, *NH*), 8. 24 (1H, d, *J*= 8.0 Hz, *ArH*), 7.52 (1H, dd, *J*= 8.0; 1.4, *ArH*), 7.42 – 7.46 (1H, m, *ArH*), 7.32 (2H, d, *J*= 7.2 Hz, *ArH*), 7.21 – 7.27 (7H, m, *ArH*), 7.15 – 7.21 (2H, m, *ArH*), 7.10 (1H, td, *J*= 6.5; 1.0 Hz, *ArH*), 4.70 – 4.76 (1H, m, *CH*), 4.47 – 4.53 (1H, m, *CH*), 3.10 – 3.15 (2H, m, CH_2), 2.88 – 3.02 (2H, m, CH_2), 1.98 (3H, s, CH_3). ^{13}C NMR (DMSO-*d6*, 101 MHz) 172.67, 171.12, 168.17, 168.06, 138.26, 138.18, 137.42, 131.62, 129.13, 128.13, 128.01, 126.42, 126.25, 122.49, 121.78, 120.41, 54.48, 53.59, 36.48, 24.58. HR-MS (ESI): calcd for $C_{27}H_{27}N_3O_5$+Na: 496.1848 found 496.1840.

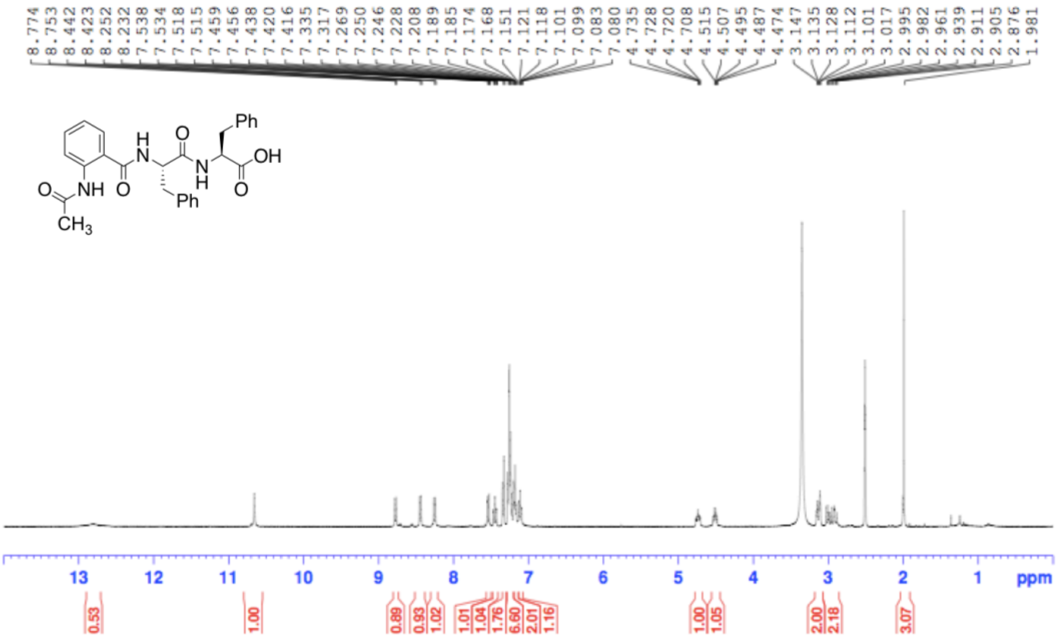

Figure S 19 ^{1}H NMR of (2-acetamidobenzoyl)-L-phenylalanyl-L-phenylalanine **1**

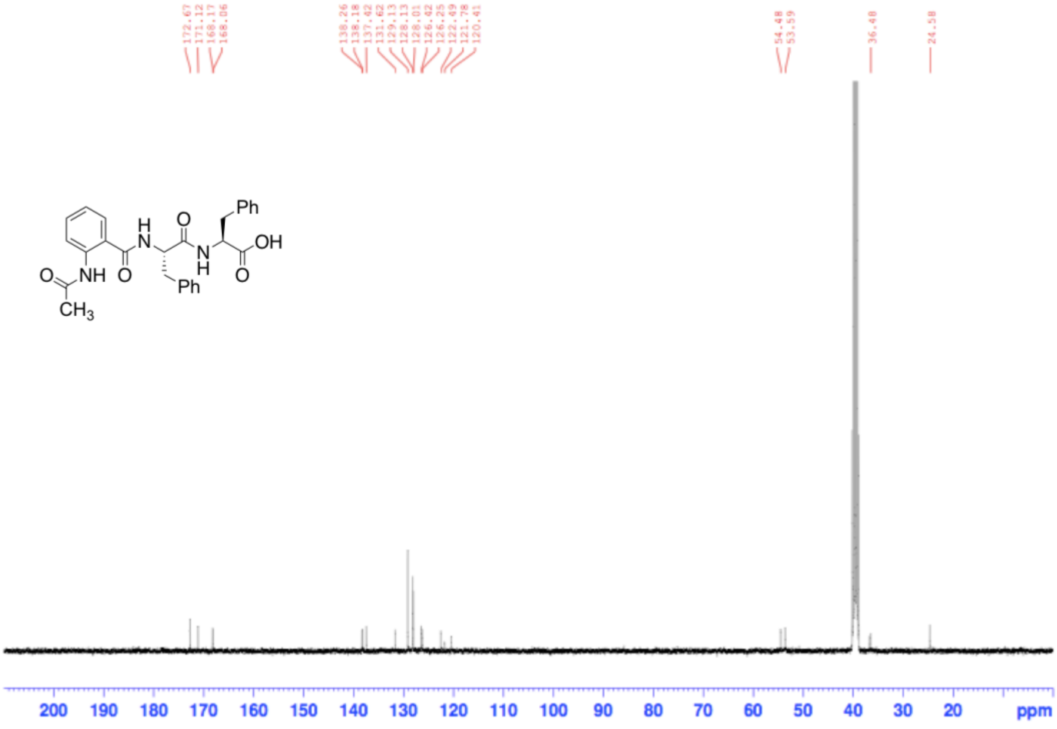

Figure S 20 ^{13}C NMR of (2-acetamidobenzoyl)-L-phenylalanyl-L-phenylalanine **1**

(2-Benzamidobenzoyl)-L-phenylalanyl-L-phenylalanine 2

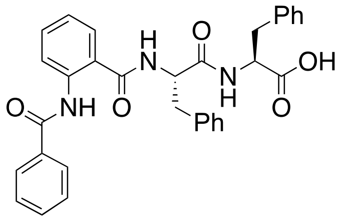

According to **GP 4,** the methyl (2-benzamidobenzoyl)-L-phenylalanyl-**L**-phenylalaninate (500 mg, 0.9 mmol) was hydrolyzed with LiOH (2.0 equivalent) to give the hydrogelators **2** as a white fluffy solid in quantitative yield (470 mg).

M.p. = 200.8-201.0 °C. IR Max: 3287,3063, 2927, 1744, 1657, 1590, 1521, 1449, 1394, 1302, 1250, 1173, 1110, 902, 747, 697. ^{1}H NMR (DMSO-*d6*, 400 MHz) 12.09 (1H, s, *NH*), 8.97 (1H, d, *J*= 8.5 Hz, *NH*), 8.61 (1H, dd, *J*= 8.4; 1.1, *ArH*) 8.45 (1H, d, J= 7.8 *NH*), 7.79 – 7.81 (2H, m, *ArH*), 7.75 (1H, dd, *J*= 7.8; 1.1 *ArH*), 7.59 – 7.63 (1H, m, *ArH*), 7.49 – 7.55 (3H, m, *ArH*), 7.34 (2H, d, *J*= 7.4 Hz, *ArH*), 7.10 – 7.23 (5H, m, *ArH*), 7.11 – 7.15 (2H, m, *ArH),* 7.04 – 7.09 (2H, m, *ArH*), 4.81 - 4.87 (1H, m, *CH*), 4.48-4.53 (1H, m, *CH*), 3.08 – 3.13 (2H, m, CH_2), 2.93 – 2.99 (2H, m, CH_2).^{13}C NMR (DMSO-*d6*, 101 MHz) 172.63, 170.88, 168.51, 164.30, 139.24, 138.14, 137.35, 134.44, 132.40, 132.02, 129.15, 129.10, 128.85, 128.50, 128.06, 127.96, 126.89, 126.35, 126.23, 122.65, 119.97, 119.84, 54.48, 53.45, 36. 85, 36.50. HR-MS (ESI): calcd for $C_{32}H_{29}N_3O_5$+Na: 558.2005 found 558.1994

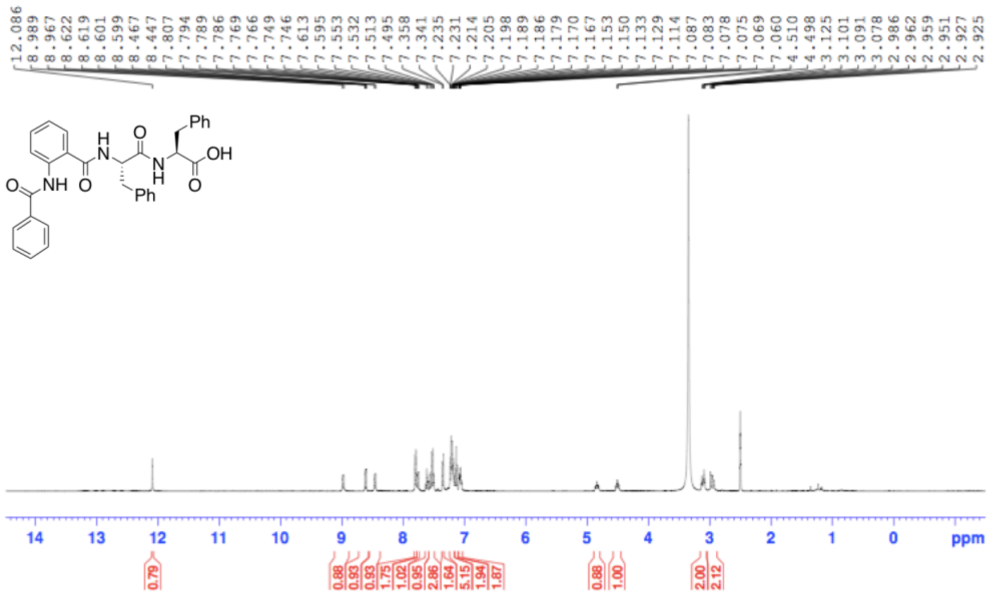

Figure S 21 ^{1}H NMR of (2-benzamidobenzoyl)-L-phenylalanyl-L-phenylalanine **2**

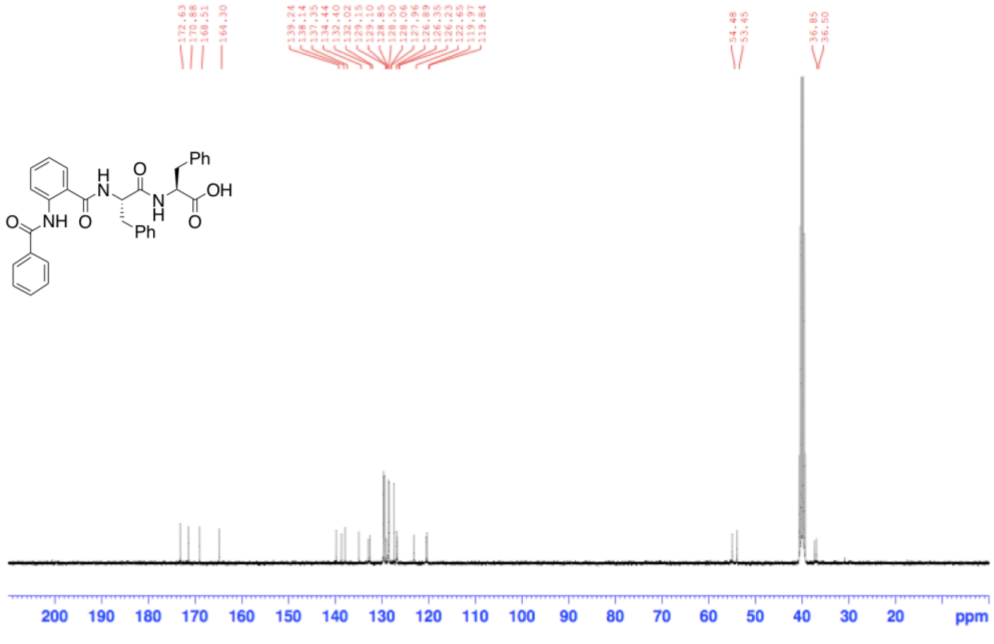

Figure S 22 ^{13}C NMR of (2-benzamidobenzoyl)-L-phenylalanyl-L-phenylalanine **2**

(2-(2-Naphthamido)benzoyl)-L-phenylalanyl-L-phenylalanine 3

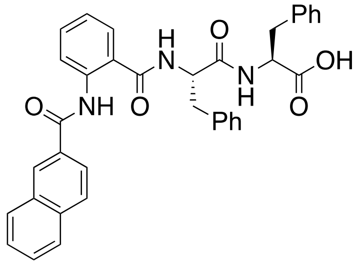

The methyl (2-(2-naphthamido)benzoyl)-L-phenylalanyl-L-phenylalaninate (700 mg, 1.2 mmol) was hydrolyzed according to **GP4** to provide hydrogelator **3** as white powder in quantitative yield (683 mg).

M.p. = 231.6 °C. IR Max 3269, 1737, 1654, 1589, 1520, 1440, 1302, 1170, 1107, 1031, 913, 748, 697. ^{1}H NMR (DMSO-d6, 400 MHz) 12.80 (1H, bs, *OH*), 12.22 (1H, s, *NH*), 9.00 (1H, d, *J*= 8.9 Hz, *NH*), 8.63 (1H, dd, *J*= 8.3; 1.2, *ArH*) 8.47 (1H, d, *J*= 7.9, *NH*), 8.43 (1H, d, *J*= 1.3, *ArH*), 7.99 – 8.09 (3H, m, *ArH*), 7.85 (1H, dd, *J*= 8.3; 1.7 *ArH*), 7.78 (1H, dd, *J*= 7.9; 1.3 Hz, *ArH*), 7.55 – 7.69 (3H, m, *ArH*), 7.35 (1H, dd, *J*= 8.3 Hz, *ArH*), 7.15 – 7.24 (5H, m, *ArH*), 7.01 – 7.09 (2H, m, *ArH*), 6.95 – 6.99 (1H, m, *ArH*), 4.83 - 4.89 (1H, m, *CH*), 4.547 - 4.52 (1H, m, *CH*), 3.06 – 3.17 (2H, m, *CH$_2$*), 2.91 – 3.00 (2H, m, *CH$_2$*). ^{13}C NMR (DMSO-*d6*, 101 MHz) 172.65, 170.91, 168.50, 164.43, 139.22, 138.15, 137.36, 134.40, 132.39, 132.20, 131.86, 129.15, 129.10, 129.04, 128.58, 128.52, 128.11, 128.01, 127.96, 127.79, 127.68, 127.03, 126.26, 126.22, 123.22, 122.75, 120.09, 54.47, 53.46, 36.88, 36.50. MS (ESI): calcd for $C_{36}H_{31}N_3O_5$+Na: 608.2161 found 608.2153.

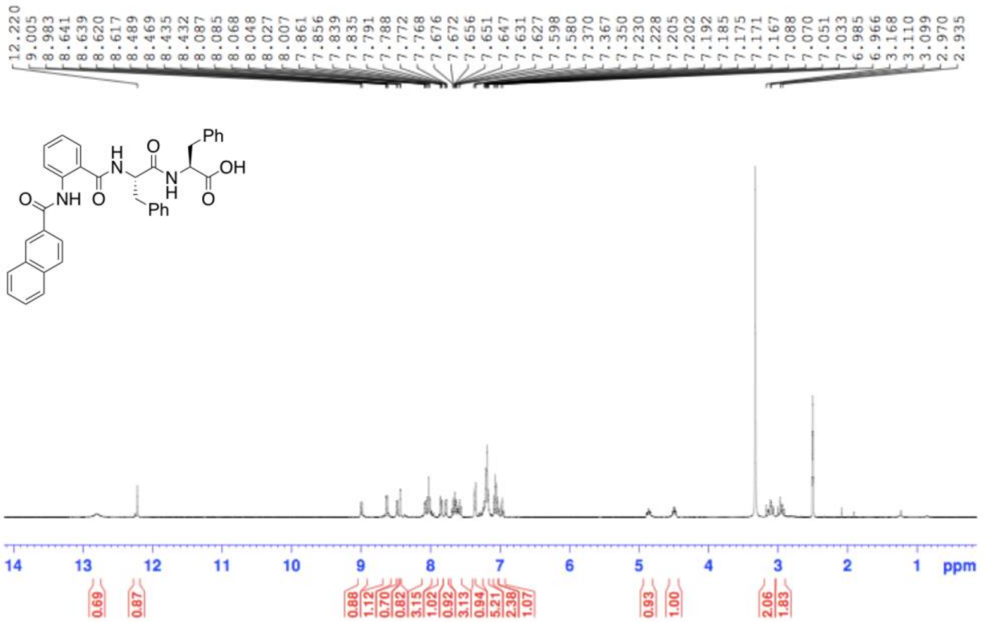

Figure S 23 ^{1}H NMR of (2-(2-naphthamido)benzoyl)-L-phenylalanyl-L-phenylalanine **3**

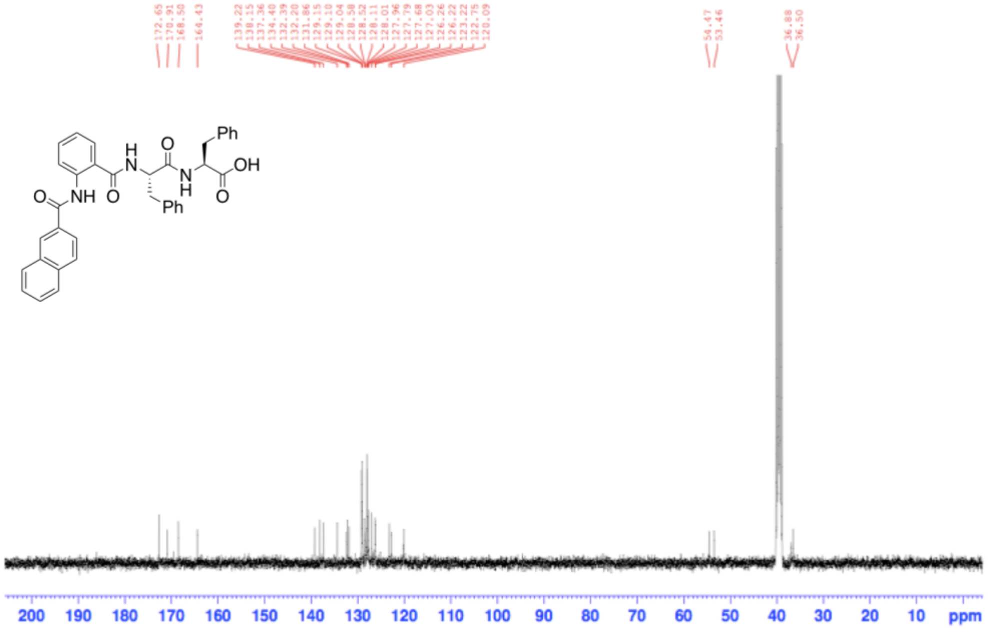

Figure S 24 ^{13}C NMR of (2-(2-naphthamido)benzoyl)-L-phenylalanyl-L-phenylalanine **3**

(2-(Naphthalene-1-sulfonamido)benzoyl)-L-phenylalanyl-L-phenylalanine 4

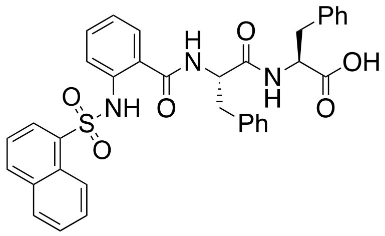

According to **GP4**, methyl (2-(naphthalene-1-sulfonamido)benzoyl)-L-phenylalanyl-L-phenylalaninate (400 mg, 0.6 mmol) was hydrolyzed to obtained hydrogelator **4** in quantitative yield (390 mg).

M.p.= 101.3-101.6°C. IR Max 3309, 3030, 2928, 2631, 2321, 1719, 1631, 1508, 1329, 1262, 1159, 979,928, 802, 747, 698. ^{1}H NMR (DMSO-*d6*, 400 MHz) 12.73 (1H, bs, *OH*), 12.15 (1H, s, *NH*), 8.83 (1H, d, *J*= 7.51 Hz, *NH*), 8.55 (1H, d, *J*= 7.51, *NH*), 8.43 (1H, d, *J*= 8.64, *ArH*), 8.30 (1H, d, *J*= 7.51, *ArH*), 8.20 (1H, d, *J*= 8.26, *ArH*), 8.02 (1H, d, *J*= 8.26, *ArH*), 7.67 (1H, d, *J*= 7.91, *ArH*), 7.62 (1H, t, *J*= 7.84, *ArH*), 7.59 (1H, t, *J*= 7.29, *ArH*), 7.48 (1H, d, *J*= 7.62, *ArH*) 7.24 – 7.35 (1H, m, *ArH*), 7.14 (3H, m, *ArH*), 6.96 – 6.99 (1H, m, *ArH*), 4.76 -4.82 (1H, m, *CH*), 4.53-4.59 (1H, m, *CH*), 3.09 – 3.20 (2H, m, CH_2), 2.91 – 3.05 (2H, m, CH_2). ^{13}C NMR (DMSO-*d6*, 101 MHz) 172.76, 170.91, 168.04, 137.99, 137.46, 134.89, 133.72, 132.74, 130.32, 129.20, 129.16, 129.08, 128.80, 128.44, 128.21, 128.08, 127.08, 127.03, 126.51, 126.32, 124.41, 123.37, 122.25, 118.09, 116.99, 54.58, 53.65, 36.76, 36.63. HR-MS (ESI): calcd $C_{35}H_{31}N_3O_{6S}$+H: 622.2012 found 622.2007.

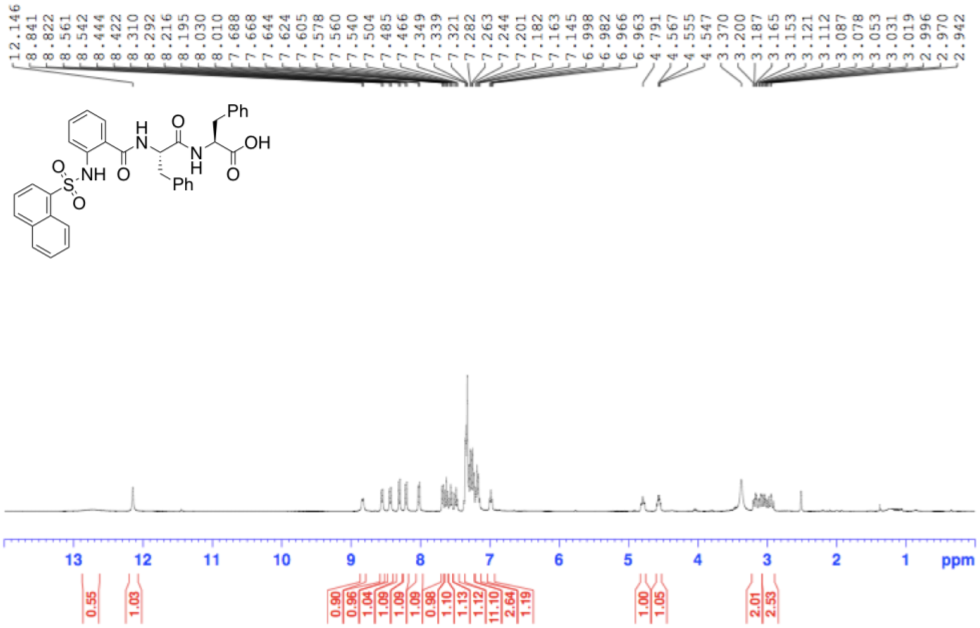

Figure S 25 ¹H NMR (2-(naphthalene-1-sulfonamido)benzoyl)-L-phenylalanyl-L-phenylalanine **4**

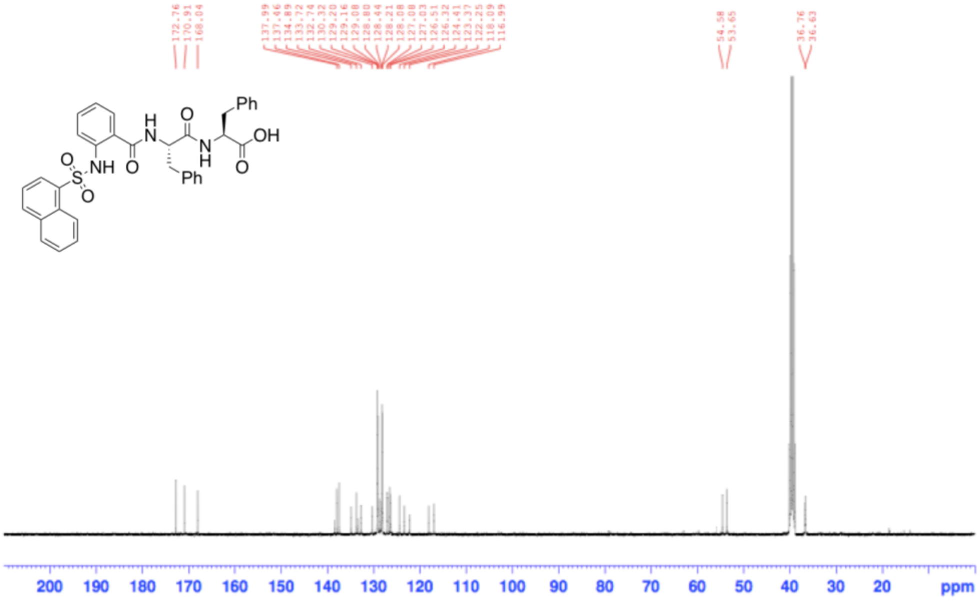

Figure S 26 ¹³C NMR (2-(naphthalene-1-sulfonamido)benzoyl)-L-phenylalanyl-L-phenylalanine **4**

(2-Benzamido-5-fluorobenzoyl)-L-phenylalanyl-L-phenylalanine 5

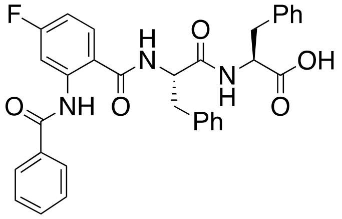

According to **GP4**, methyl (2-benzamido-5-fluorobenzoyl)-L-phenylalanyl-L-phenylalaninate (600 mg) was hydrolyzed with LiOH (2.0 equivalent) to give hydrogelator **5** as a white solid in quantitative yield (585 mg). M.p.= 235.2 °C. IR Max: 3297, 2923, 2321, 2099, 1717, 1657, 1599, 1522, 1413, 1297, 1239, 1108, 1028, 947, 874, 830, 746, 696.

^{1}H NMR (DMSO-*d6*, 400 MHz) 12.79 (1H, bs, *OH*), 11.88 (1H, s, *NH*), 9.05 (1H, d, *J*= 8.65 Hz, *NH*), 8.64 (1H, d, *J*= 8.41, *NH*) 8.58 – 8.63 (1H, dd, *J*= 9.24; 5.48 Hz, *ArH*), 8.48 (1H, d, *J*= 8.08 Hz, *NH*), 7.79 (2H, dd, *J*= 6.9; 1.1Hz, *ArH*), 7.58 – 7.62 (2H, m, *ArH*), 7.41 – 7.54 (4H, m, *ArH*), 7.43 – 7.45 (1H, m, *ArH*), 7.32 (2H, d, *J*= 7.14, *ArH*), 7.33 (2H, d, *J*= 6.92, *ArH*), 7.03 – 7.24 (10H, m, *ArH*), 4.79 -4.87 (1H, m, *CH*), 4.46-4.53 (1H, m, *CH*), 3.07 – 3.17 (2H, m, *CH_2*), 2.88 – 2.99 (2H, m, *CH_2*). ^{13}C NMR (DMSO-*d6*, 101 MHz) 172.65, 170.72, 167.25, 164.28, 158.05, 155.66, 138.03, 137.42, 135.66, 134.25, 132.09, 129.11, 129.11, 128.87, 128.06, 127.98, 126.91, 126.35, 126.28, 122.21, 122.13, 121.64, 119.19, 118.98, 115.19, 114.95, 54.94, 53.51, 36.88, 36.45. HR-MS (ESI): calcd $C_{32}H_{28}FN_3O_5$+Na: 576.1911 found 576.1902.

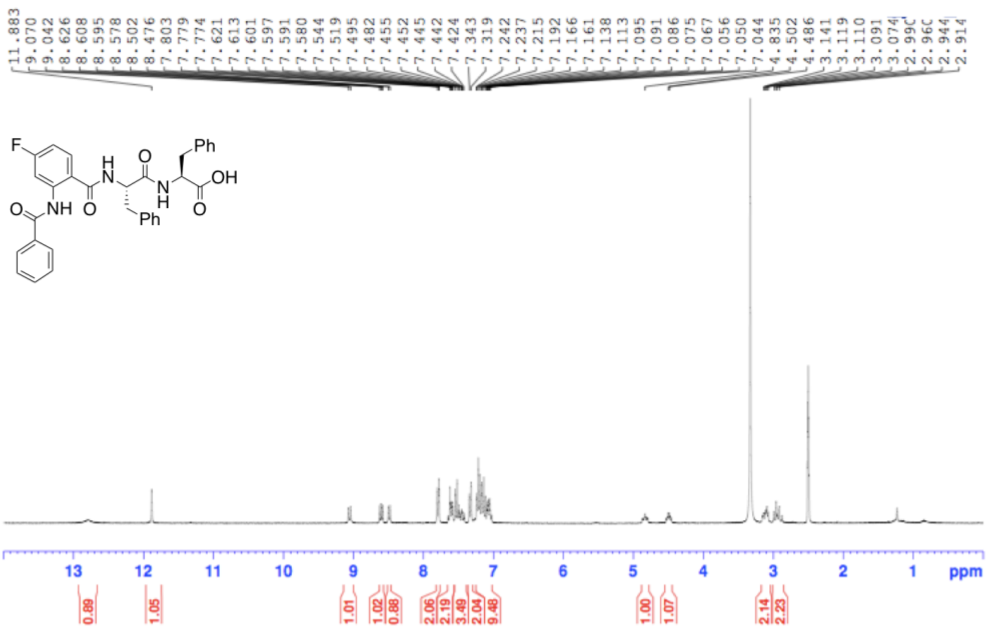

Figure S 27 ^{1}H NMR of (2-benzamido-5-fluorobenzoyl)-L-phenylalanyl-L-phenylalanine **5**

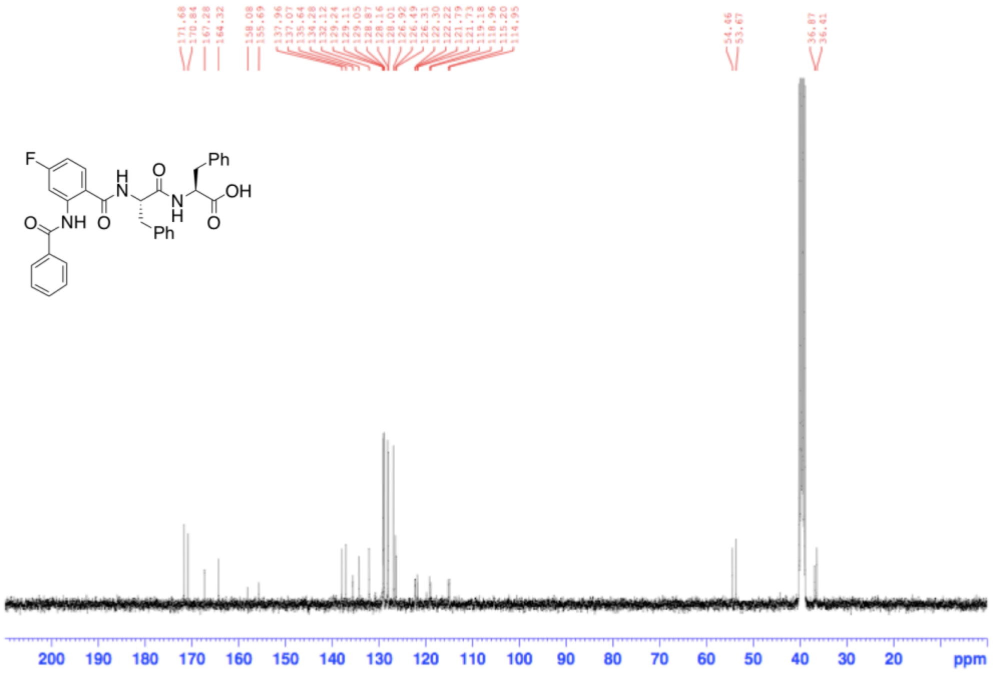

Figure S 28 ^{13}C NMR of (2-benzamido-5-fluorobenzoyl) -L-phenylalanyl-L-phenylalanine **5**

(2-Benzamido-5-methylbenzoyl)-L-phenylalanyl-L-phenylalanine 6

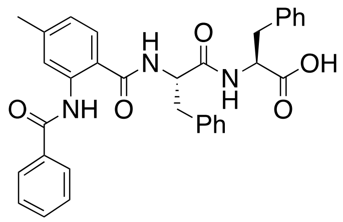

Methyl (2-benzamido-5-methylbenzoyl)-L-phenylalanyl-L-phenylalaninate (550 mg, 1.0 mmol) was hydrolyzed using LiOH (2.0 equivalent), according to **GP4**, to give hydrogelator **6** in quantitative yield.

IR Max: 3307, 1748, 1658, 1587, 1524, 1456, 1399, 1309, 1251, 1176, 1111, 828, 697. ^{1}H NMR (DMSO-*d6*, 400 MHz) 12.81 (1H, bs, *OH*), 11.94 (1H, s, *NH*), 8.90 (1H, d, *J*= 8.7 Hz, *NH*), 8.50 (1H, d, *J*= 8.5 Hz, *NH*), 8.45 (1H, d, *J*= 7.8 Hz, *ArH*), 7.77 – 7.79 (2H, m, *ArH*), 7.57 – 7.60 (2H, m, *ArH*), 7.49 – 7.52 (2H, m, *ArH*), 7.33 – 7.37 (3H, m, *ArH*), 7.05 – 7.23 (9H, m, *ArH*), 4.82 – 4.88 (1H, m, *CH*), 4.47 – 4.53 (1H, m, *CH*), 3.08 – 3.14 (2H, m, CH_2), 2.91 – 2.98 (2H, m, CH_2), 2.33 (3H, s, CH_3). ^{13}C NMR (DMSO-*d6*, 101 MHz) 172.65, 170.91, 168.48, 164.10, 138.10, 137.39, 136.78, 134.53, 132.76, 131.92, 131.70, 129.16, 129.10, 128.83, 128.78, 128.07, 127.94, 126.85, 126.36, 126.24, 119.95, 119.88, 54.32, 53.50, 36.94, 36.49, 20.40. HR-MS (ESI): calcd $C_{33}H_{31}N_3O_5$+Na: 572.2161 found 572.2153.

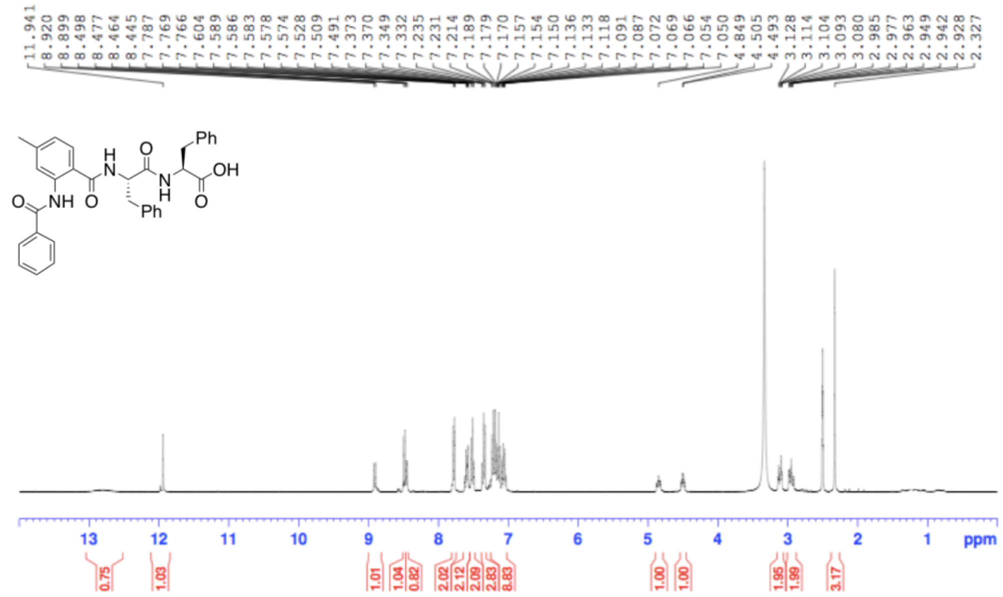

Figure S 29 ^{1}H NMR of (2-benzamido-5-methylbenzoyl)-L-phenylalanyl-L-phenylalanine **6**

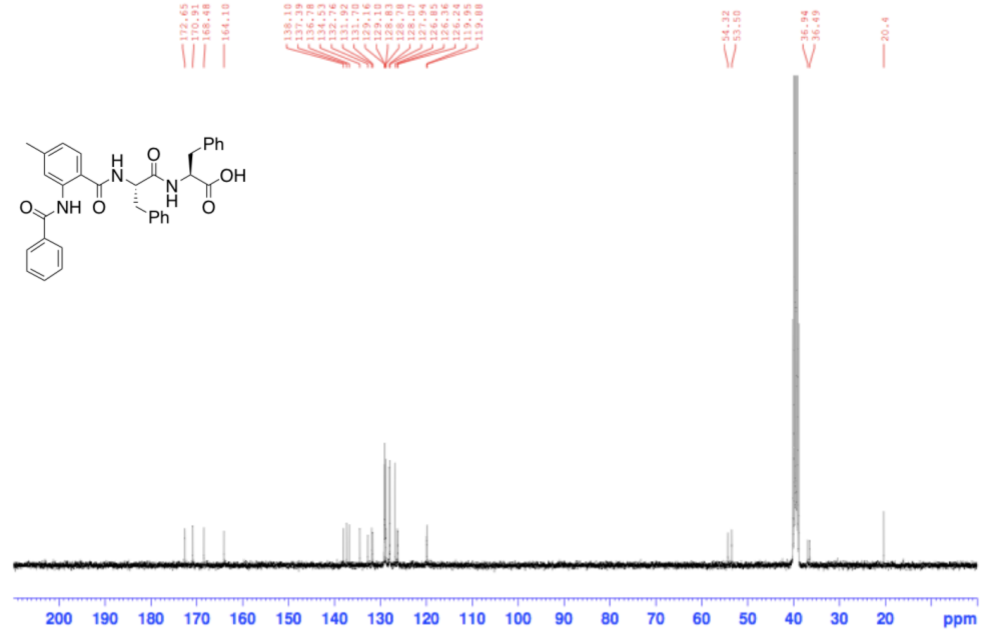

Figure S 30 ^{13}C NMR of (2-benzamido-5-methylbenzoyl)-L-phenylalanyl-L-phenylalanine **6**

(2-(2-((2-(((*R*)-1-Carboxy-2-phenylethyl)carbamoyl)phenyl)carbamoyl)benzamido)benzoyl)-L-phenylalanine 7

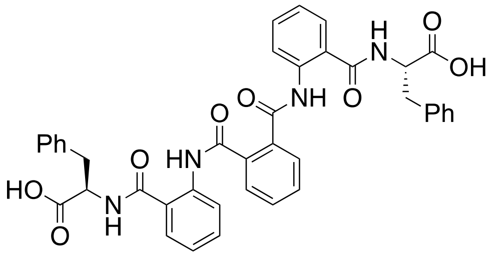

Accodring to **GP4**, methyl (2-(2-((2-(((*R*)-1-methoxy-1-oxo-3-phenylpropan-2-yl)carbamoyl)phenyl) carbamoyl)-benzamido)-benzoyl)-L-phenylalaninate (160 mg, 0.22 mmol) was reacted with LiOH (4.0 equivalents) to provide compound **7** as pure off-white powder in quantitative yield (155 mg).

IR Max= 3332, 3308, 2954, 1745, 1679, 1630, 1600, 1520, 1440, 1357, 1300, 1215, 1179, 982, 930, 743, 700. M.p.= 195.1 – 195.4 °C. ^{1}H NMR (DMSO-*d6*, 400 MHz) 11.58 (2H, s, *NH*), 9.18 (2H, d, *J*= 8.0 Hz, *NH*), 8.43 (2H, d, *J*= 8.4 Hz, *ArH*), 7.73 – 7.76 (2H, m, *ArH*), 7.66 – 7.69 (2H, m, *ArH*), 7.50 – 7.60 (4H, m, *ArH*), 7.27 (4H, s, *ArH*), 7.25 (4H, d, *J*= 7.3 Hz, *ArH*), 7.15 – 7.20 (5H, m, *ArH*), 4.64 – 4.72 (2H, m, *CH*), 3.02 – 3.61 (4H, m, CH_2). ^{13}C NMR (DMSO-*d6*, 101 MHz)171.62, 168.43, 165.87, 139.04, 137.44, 136.43, 132.41, 130.87, 129.05, 128.30, 128.23, 127.26, 126.55, 122.96, 120.33, 120.00, 54.03, 36.09. HR-MS (ESI): calcd $C_{40}H_{34}N_4O_8$ 721.2274 found 721.2267.

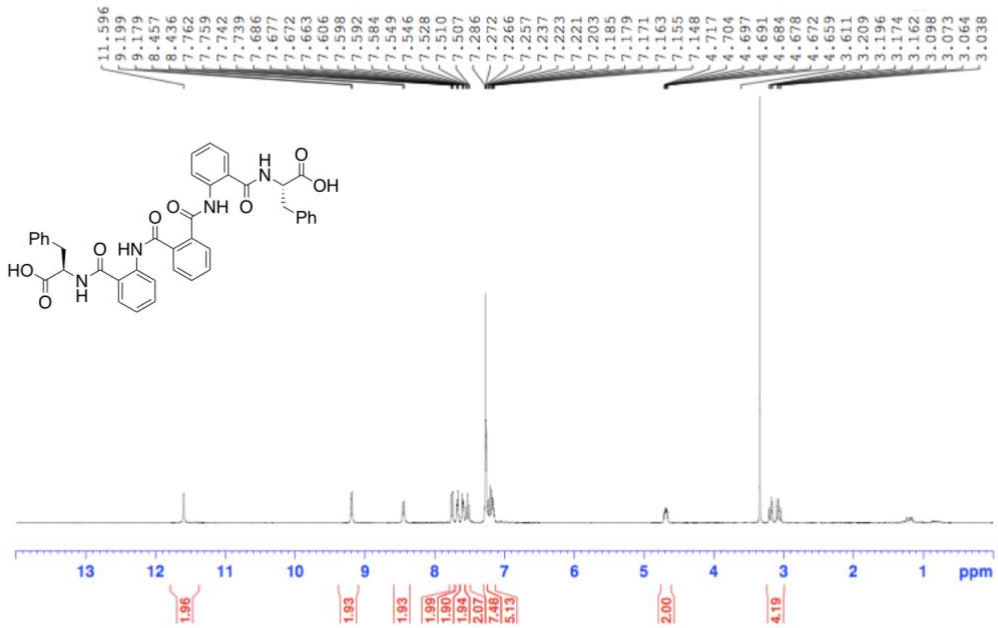

Figure S 31 ^{1}H NMR of (2-(2-((2-(((R)-1-carboxy-2-phenylethyl)carbamoyl)phenyl)carbamoyl)benzamido)benzoyl)-L-phenylalanine **7**

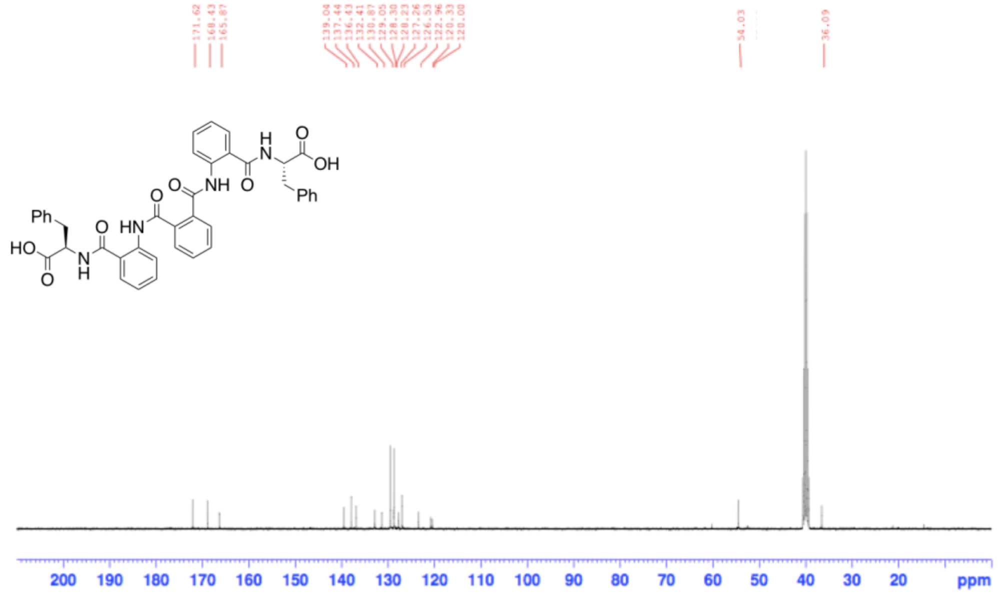

Figure S 32 ^{13}C NMR of (2-(2-((2-(((R)-1-carboxy-2-phenylethyl)carbamoyl)phenyl)carbamoyl)benzamido)benzoyl)-L-phenylalanine **7**

(2-(3-((2-(((*R*)-1-Carboxy-2-phenylethyl)carbamoyl)phenyl)carbamoyl)benzamido)benzoyl)-L-phenylalanine 8

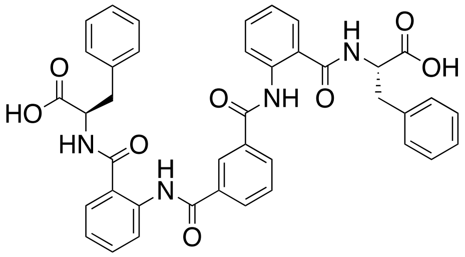

According to **GP4**, methyl (2-(3-((2-(((*R*)-1-methoxy-1-oxo-3-phenylpropan-2yl)carbamoyl)phenyl)carbamoyl) -benzamido)benzoyl)-L-phenylalaninate **13** (250 mg) was reacted with LiOH (4.0 equivalents), according to **GP 4**, to provide hdyrogleator **8** as off-white powder (250 mg).

IR Max= 3304, 2057, 2006, 1738, 1631, 1596, 1515, 1443, 1305, 1217, 845, 753, 697. M.p.= 200.7 – 201.1 °C. ^{1}H NMR (DMSO-*d6*, 400 MHz) 12.18 (2H, s, *NH*), 9.14 (2H, d, *J*= 8.2 Hz, *NH*), 8.60 (2H, d, *J*= 8.2 Hz, *ArH*), 8.45 (1H, s, *ArH*), 8.01 (2H, d, *J*= 6.9 Hz, *ArH*), 7.78 – 7.81 (3H, m, *ArH*), 7.60 (2H, t, *J*= 7.5 Hz, *ArH*), 7.30 – 7.32 (4H, m, *ArH*), 7.20 -7.27 (6H, m, *ArH*), 7.07 - 7.11 (2H, m, *ArH*), 4.73 – 4.79 (2H, m, *CH*), 3.04 – 3.27 (4H, m, CH_2). ^{13}C NMR (DMSO-*d6*, 101 MHz) 172.69, 168.67, 163.63, 138.93, 137.91, 135.22, 132.53, 129.77, 129.71, 129. 09, 128.40, 128.18, 126.67, 126.43, 123.20,120.56,120.36, 54.02, 36.25. HR-MS (ESI): calcd $C_{40}H_{34}N_4O_8$ 721.2274 found 721.2265.

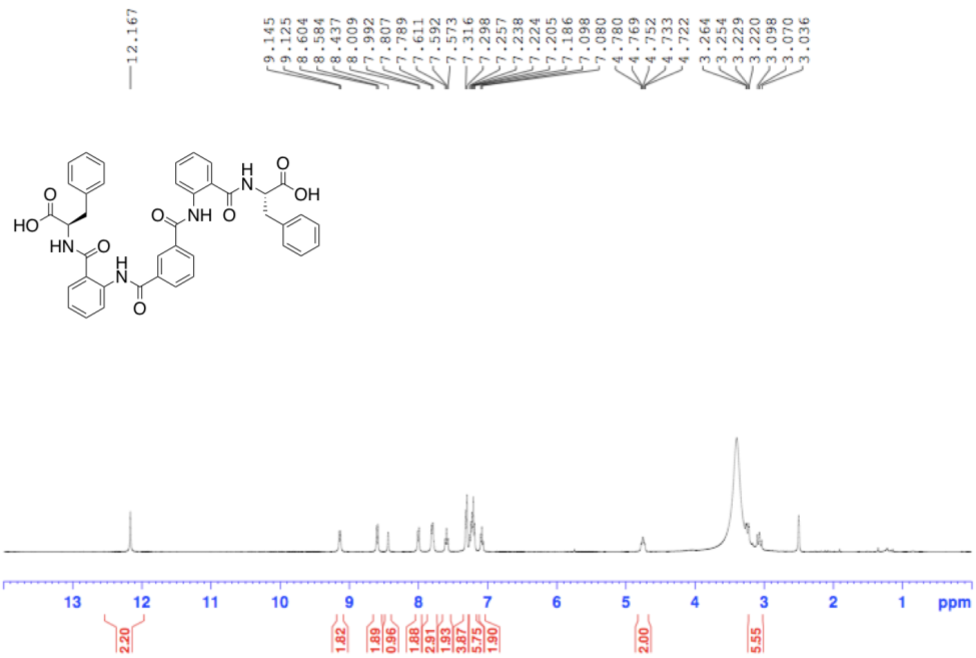

Figure S 33 ^{1}H NMR of (2-(3-((2-(((R)-1-carboxy-2-phenylethyl)carbamoyl)phenyl)carbamoyl)benzamido)benzoyl)-L-phenylalanine **8**

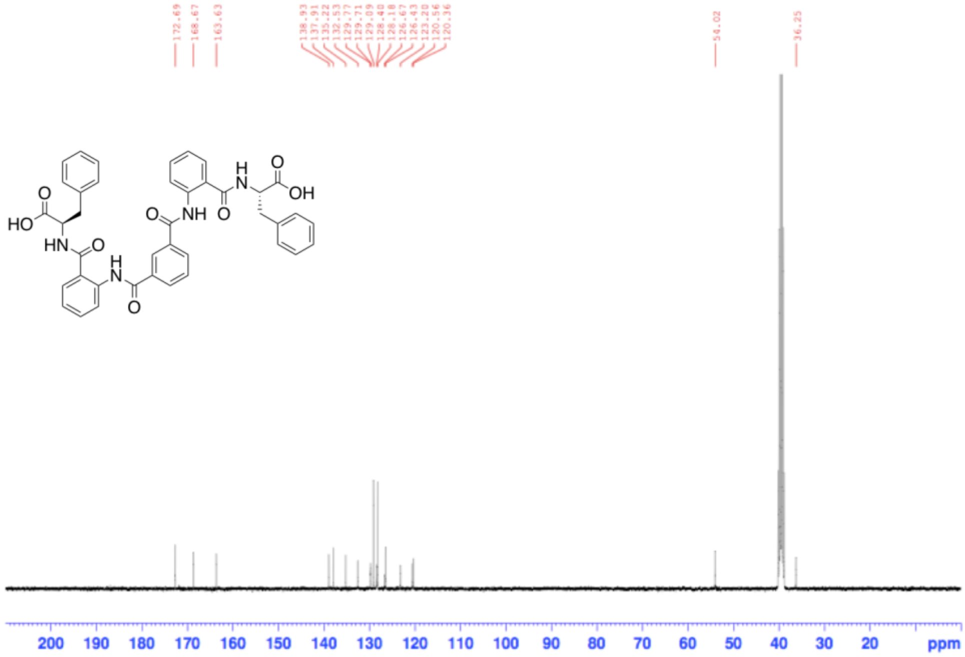

Figure S 34 ^{13}C NMR of (2-(3-((2-(((R)-1-carboxy-2-phenylethyl)carbamoyl)phenyl)carbamoyl)benzamido)benzoyl)-L-phenylalanine **8**

(2-(4-((2-(((*R*)-1-Carboxy-2-phenylethyl)carbamoyl)phenyl)carbamoyl)benzamido)benzoyl)-L-phenylalanine 9

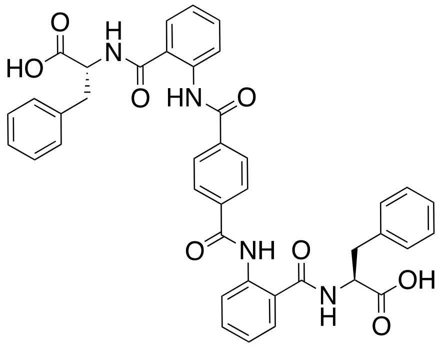

According to **GP4**, Methyl (2-(4-((2-(((*R*)-1-methoxy-1-oxo-3-phenylpropan-2-yl)carbamoyl)phenyl)-carbamoyl)-benzamido)-benzoyl)-L-phenylalaninate (900 mg, 1.2 mmol) was hydrolyzed using LiOH (4.0 equivalents) to obtain hydrogelator **9** in quantitative yield (865 mg).

M.p. = 236.5 -236.8 °C. IR Max:3310, 2921, 2603, 2519, 2112, 1930, 1717, 1660, 1594, 1522, 1444, 1300, 1165, 1125, 862, 754, 681. ^{1}H NMR (DMSO-*d6*, 400 MHz) 12.96 (2H, bs, *OH*), 12.10 (2H, bs, *NH*), 9.16 (2H, d, *J*= 8.2 Hz*, NH?*), 8.60 (2H, d, *J*= 8.2 Hz*, ArH*), 7.96 (4H, s, *ArH*), 7.78 (2H, t, *J*= 6.7 Hz, *ArH*), 7.59 (2H, t, *J*= 7.7 Hz, *ArH*), 7.32 (4H, d, *J*= 7.6, *ArH*), 7.24 (6H, t, *J*= 7.6 Hz, *ArH*), 7.10 (2H, t, *J*= 7.7 Hz, *ArH*), 4.76 – 4.78 (2H, m, *CH*), 3.06 – 3. 25 (4H, m, *CH$_2$*). ^{13}C NMR (DMSO-*d6*, 101 MHz) 172.66, 168.65, 163.54, 138.86, 137.96, 137.38, 132.48, 129.10, 128.36, 128.15, 127.56, 126.41, 123.17,120.33, 54.11, 36.26. HR-MS (ESI): calcd $C_{40}H_{34}N_4O_8$ 721.2274 found 721.2265.

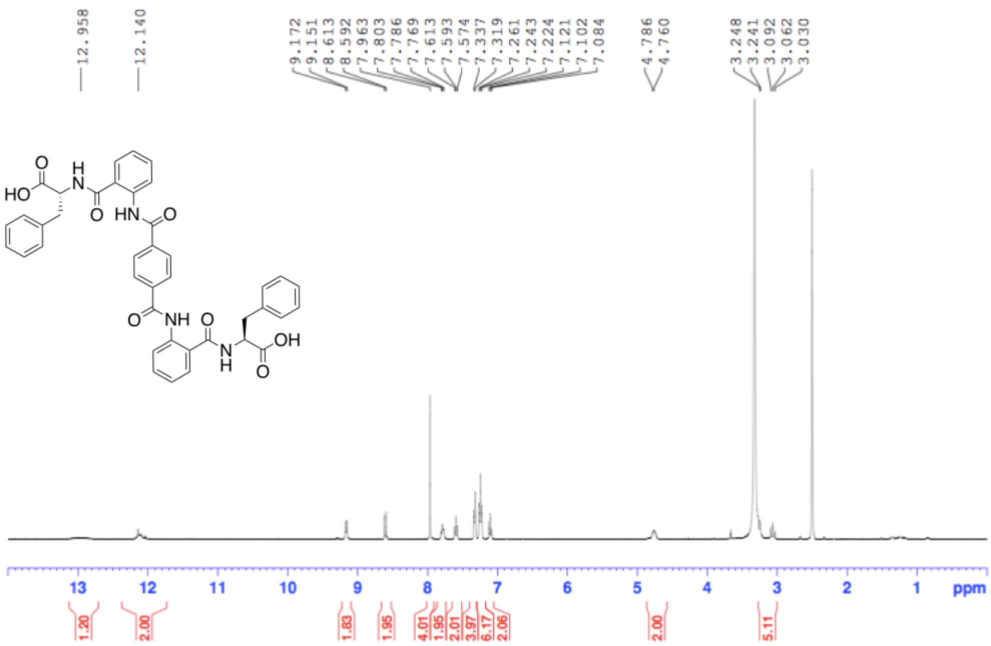

Figure S 35 ¹H NMR of (2-(4-((2-(((R)-1-carboxy-2-phenylethyl)carbamoyl)phenyl)carbamoyl)benzamido)benzoyl) -L-phenylalanine **9**

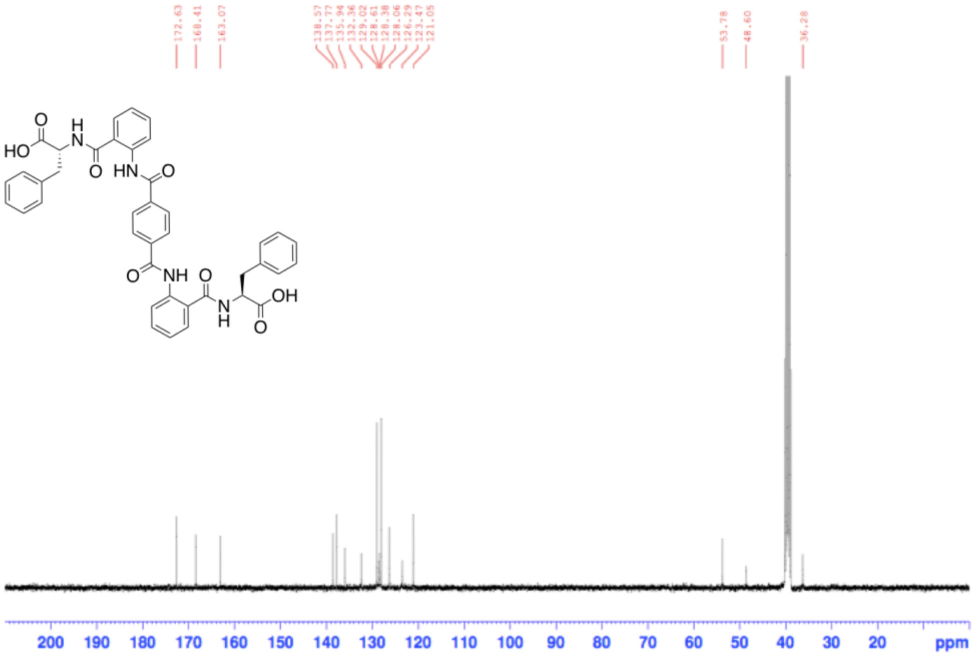

Figure S 36 ¹³C NMR of (2-(4-((2-(((R)-1-carboxy-2-phenylethyl)carbamoyl)phenyl)carbamoyl)benzamido)benzoyl)-L-phenylalanine **9**

(2-(2-((2-(((*R*)-1-Carboxy-2-phenylethyl)carbamoyl)phenyl)amino)-2-oxoacetamido)-benzoyl)-L-phenylalanine 10

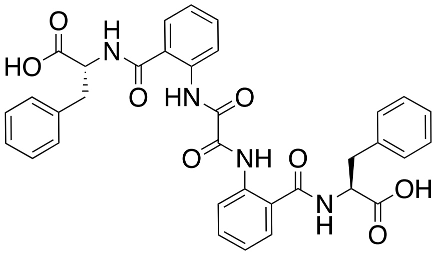

Methyl (2-(2-((2-(((*R*)-1-methoxy-1-oxo-3-phenylpropan-2-yl)carbamoyl)phenyl)amino)-2-oxoacetamido)benzoyl) -L-phenylalaninate (800 mg, 1.1 mmol) was hydrolyzed according to **GP4** to obtain the hydrogelator **10** in quantitative yields (700 mg).

M.p= 280.9 °C. IR Max 3024, 2690, 2107, 1691, 1630, 1582, 1512, 1448, 1298, 1236, 1166, 1112, 911, 869, 802, 749, 697. ^{1}H NMR (DMSO-*d6*, 400 MHz) 12.90 (2H, bs, *OH*), 12.57 (2H, s, *NH*), 9.06 (2H, d, *J*= 7.4 *Hz*, *NH*), 8.54 (2H, d, *J*= 7.4 *Hz*, *ArH*), 7.79 (2H, d, *J*= 7.4 *Hz*, *ArH*), 7.59 (2H, t, *J*= 7.2 *Hz*, *ArH*), 7.23 – 7.33 (10H, m, *ArH*), 7.14 – 7.15 (2H, m, *ArH*), 4.66 - 4.78 (2H, m, *CH*), 3.05 – 3.26 (4H, m, CH_2). ^{13}C NMR (DMSO-*d6*, 101 MHz) 172.72, 167.78, 157.73, 137.96, 137.33, 132.43, 129.04, 128.47, 128.21, 126.38, 123.95, 120.90, 120.08, 54.06, 36.15. HR-MS (ESI): calcd $C_{34}H_{30}N_4O_8$+Na: 645.1961 found 645.1965.

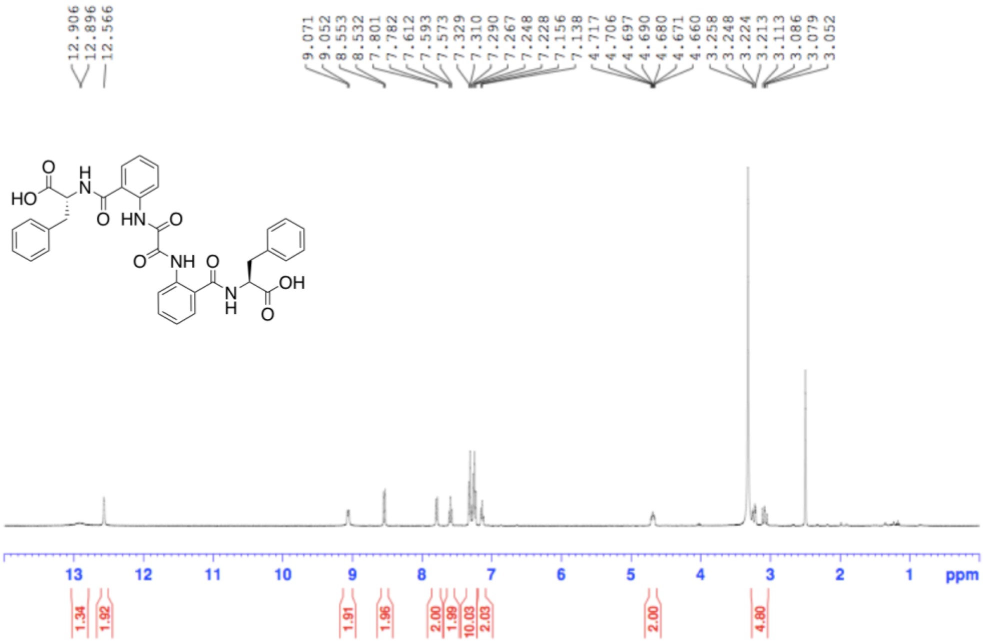

Figure S 37 ¹H NMR of (2-(2-((2-(((R)-1-carboxy-2-phenylethyl)carbamoyl)phenyl)amino)-2-oxoacetamido)-benzoyl)-L-phenylalanine **10**

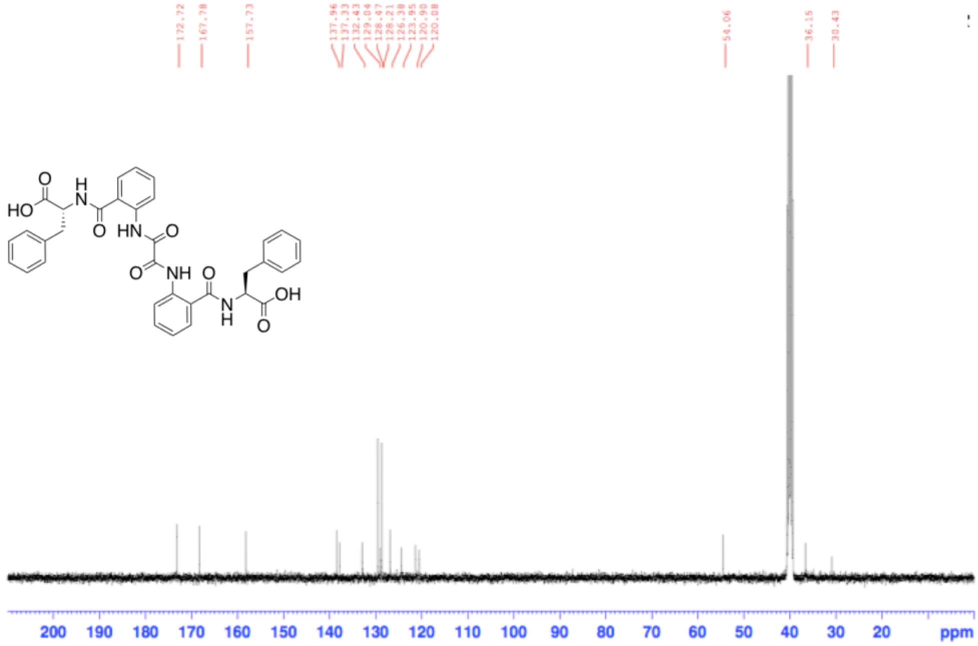

Figure S 38 ¹³C NMR of (2-(2-((2-(((R)-1-carboxy-2-phenylethyl)carbamoyl)phenyl)amino)-2-oxoacetamido)-benzoyl)-L-phenylalanine **10**

(2-(3-((2-((((*R*)-1-Carboxy-2-phenylethyl)amino)(hydroxy)methyl)phenyl)carbamoyl)-5-((2-(((*R*)-1-carboxy-2-phenylethyl)carbamoyl)phenyl)carbamoyl)benzamido)benzoyl)-L-phenylalanine 11

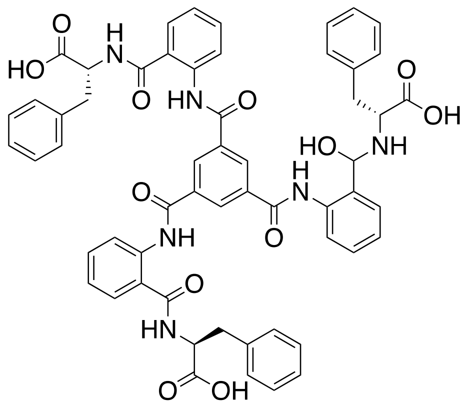

According to **GP4**, the corresponding methyl ester (400 mg, 0.4 mmol) was reacted with LiOH (4.0 equivalents) to obtained the C_3-symmetric hydrogelator **11** in quantitative yields (380 mg).

IR Max: 3295, 2923, 2603, 2343, 2113, 1721, 1638, 1587, 1509, 1444, 1302, 1220, 957, 877, 752, 697. M.p.= 222.6 – 222.9 °C. ^{1}H NMR (DMSO-*d6*, 400 MHz) 12.89 (3H, bs, *OH*)), 12.20 (3H, s, *NH*), 9.08 (3H, d, *J*= 7.8 Hz, *NH*), 8.54- 8.56 (6H, m, *ArH*), 7.81 (3H, d, *J*= 7.6 Hz, *ArH*), 7.61 (3H, t, *J*= 7.6 Hz, *ArH*), 7.25 – 7.28 (9H, m, *ArH*), 7.14 (6H, t, *J*= 7.6 Hz, *ArH*), 7.01 – 7.05 (3H, m, *ArH*), 4.74 – 4.80 (3H, m, *CH*), 3.01 – 3.24 (6H, m, *CH_2*).^{13}C NMR (DMSO-*d6*, 101 MHz) 172.64, 168. 42, 163.08, 138.57, 137.78, 135.95, 132.37, 129.02, 128.62, 128.39, 128.07, 126.29, 123.47, 121.06, 48.60, 36.29. HR-MS (ESI): calcd $C_{57}H_{48}N_6O_{12}$+Na: 1031.3228 found 1031.3214

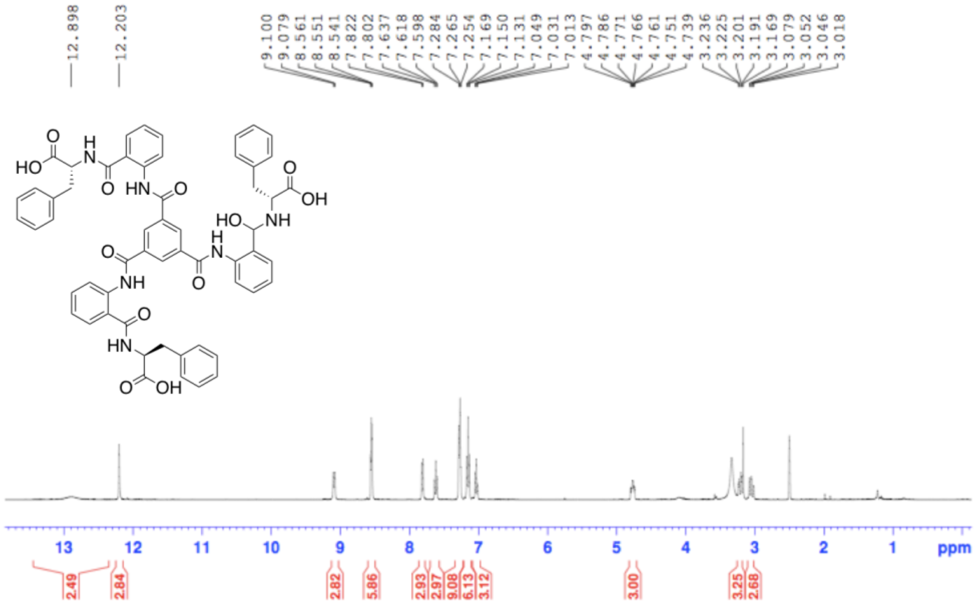

Figure S 39 (2-(3-((2-((((R)-1-carboxy-2-phenylethyl)amino)(hydroxy)methyl)phenyl)carbamoyl)-5-((2-(((R)-1-carboxy-2-phenylethyl)carbamoyl)phenyl)carbamoyl)benzamido)benzoyl)-L-phenylalanine **11**

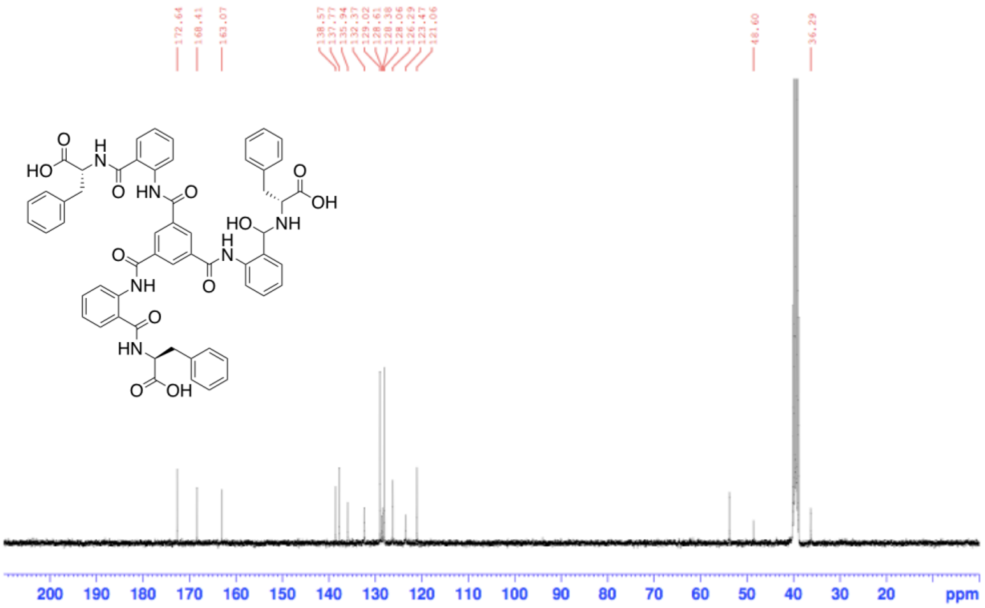

Figure S 40 (2-(3-((2-((((R)-1-carboxy-2-phenylethyl)amino)(hydroxy)methyl)phenyl)carbamoyl)-5-((2-(((R)-1-carboxy-2-phenylethyl)carbamoyl)phenyl)carbamoyl)benzamido)benzoyl)-L-phenylalanine **11**

References

1 Liu, Y. *et al.* Building Nanowires from Micelles: Hierarchical Self-Assembly of Alternating Amphiphilic Glycopolypeptide Brushes with Pendants of High-Mannose Glycodendron and Oligophenylalanine. *J Am Chem Soc* **138**, 12387-12394 (2016).

2 Anil, S. M. *et al.* Facile synthesis of 1, 4-benzodiazepine-2, 5-diones and quinazolinones from amino acids as anti-tubercular agents. *New J. Chem.* **43**, 182-187 (2019).

3 Khattab, S. N. *et al.* Synthesis and evaluation of quinazoline amino acid derivatives as mono amine oxidase (MAO) inhibitors. *Bioorganic & medicinal chemistry* **23**, 3574-3585 (2015).
